# Cocoa Consumption Alters the Global DNA Methylation of Peripheral Leukocytes in Humans with Cardiovascular Disease Risk Factors: A Randomized Controlled Trial

**DOI:** 10.1371/journal.pone.0065744

**Published:** 2013-06-26

**Authors:** Anna Crescenti, Rosa Solà, Rosa M. Valls, Antoni Caimari, Josep M. del Bas, Anna Anguera, Neus Anglés, Lluís Arola

**Affiliations:** 1 Centre Tecnològic de Nutrició i Salut (CTNS), TECNIO, Reus, Spain; 2 Unitat de Recerca en Lípids i Arteriosclerosi, CIBERDEM, Hospital Universitari Sant Joan de Reus, IISPV, Universitat Rovira i Virgili, Reus, Spain; 3 Rottapharm/Madaus, S.L., Barcelona, Spain; 4 La Morella Nuts S.A.U., Tarragona, Spain; 5 Departament de Bioquímica i Biotecnologia, Nutrigenomics Research Group, Universitat Rovira i Virgili, Tarragona, Spain; University of Kansas Medical Center, United States of America

## Abstract

DNA methylation regulates gene expression and can be modified by different bioactive compounds in foods, such as polyphenols. Cocoa is a rich source of polyphenols, but its role in DNA methylation is still unknown. The objective was to assess the effect of cocoa consumption on DNA methylation and to determine whether the enzymes involved in the DNA methylation process participate in the mechanisms by which cocoa exerts these effects in humans. The global DNA methylation levels in the peripheral blood were evaluated in 214 volunteers who were pre-hypertensive, stage-1 hypertensive or hypercholesterolemic. The volunteers were divided into two groups: 110 subjects who consumed cocoa (6 g/d) for two weeks and 104 control subjects. In addition, the peripheral blood mononuclear cells (PBMCs) from six subjects were treated with a cocoa extract to analyze the mRNA levels of the DNA methyltransferases (*DNMTs*), methylenetetrahydrofolate reductase (*MTHFR*), and methionine synthase reductase (*MTRR*) genes. Cocoa consumption significantly reduced the DNA methylation levels (2.991±0.366 vs. 3.909±0.380, p<0.001). Additionally, we found an association between the cocoa effects on DNA methylation and three polymorphisms located in the *MTHFR*, *MTRR*, and *DNMT3B* genes. Furthermore, in PBMCs, the cocoa extract significantly lowered the mRNA levels of the *DNMTs*, *MTHFR*, and *MTRR*. Our study demonstrates for the first time that the consumption of cocoa decreases the global DNA methylation of peripheral leukocytes in humans with cardiovascular risk factors. *In vitro* experiments with PBMCs suggest that cocoa may exert this effect partially via the down-regulation of *DNMTs*, *MTHFR* and *MTRR*, which are key genes involved in this epigenetic process.

**Trial Registration:**

Clinicaltrials.gov NCT00511420 and NCT00502047

## Introduction

DNA methylation is a major epigenetic process that regulates gene expression and chromatin architecture. DNA methylation occurs primarily at the 5′ position of the cytosine residues within cytosine-guanine (CpG) dinucleotides and involves the addition of a methyl group to form 5-methylcytosine (5mC) [1]. This epigenetic process is catalyzed by specific DNA methyltransferases (DNMTs). There are three main DNMTs. DNMT1 is the major maintenance enzyme that preserves existing methylation patterns following DNA replication; DNMT3a and DNMT3b, in contrast, are primarily responsible for the formation of *de novo* DNA methylation patterns [1], [2]. Other enzymes, such as the folate metabolizing enzymes methylenetetrahydrofolate reductase (MTHFR) and methionine synthase reductase (MTRR), also play an important role in DNA methylation by influencing the bioavailability of methyl groups [3], [4].

Results from various studies have suggested that alterations in DNA methylation are causally involved in the development of several human diseases, including cardiovascular disease [5], [6]. In addition, it has been shown that global DNA methylation levels of peripheral lymphocytes are associated with cardiovascular disease (CVD) and also with cardiovascular risk factors, such as inflammation, atherosclerosis and cholesterol levels [6], [7], [8]. In fact, recent data have pointed to the potential for using global DNA methylation profiles of peripheral blood leukocytes as a suitable biomarker for CVD risk [5].

An interesting and important feature of epigenetics and its role in disease development is the fact that epigenetic marks are potentially reversible and can be modified by diet and environment factors [1], [9]. Polyphenols constitute one of the largest and most ubiquitous group of phytochemicals present in fruits, vegetables, and other dietary botanicals, and they are consumed in significant amounts in the human diet [1]. Several studies performed in cells have demonstrated that these bioactive food components exhibit cancer inhibition activities by affecting epigenetic signaling pathways, including DNA methylation [2].

For instance, it has been shown that epigallocatechin-3-gallate (EGCG), the major polyphenol in green tea, and the soybean isoflavone genistein can reverse DNA hypermethylation and increase the expression of various methylated-silenced tumor suppressor (TS) genes, such as methylguanine methyltransferase, *p16^INK4a^*, human MutL Homolog 1 and retinoic acid receptor β, in different human cancer cells lines [10]–[12]. In addition to EGCG and genistein, other dietary polyphenolic compounds, such as catechin, epicatechin, caffeic acid, chlorogenic acid, and curcumin, have been shown to have similar effects on DNA methylation [10], [13], [14]. Nevertheless, the evidence for a modulating effect of dietary polyphenols on DNA methylation *in vivo* is less convincing [15]–[18].

Cocoa is a rich source of polyphenols and is a significant contributor to the total dietary intake of these compounds in humans [19], [20]. However, the effect of cocoa on DNA methylation has not been addressed previously. Our hypothesis is that cocoa polyphenols reduces global DNA methylation and that this reduction is mediated via the regulation of key genes involved in this epigenetic process.

The aim of the present study was to assess the effect of cocoa on DNA methylation and to determine whether the enzymes involved in the DNA methylation process participate in the mechanisms by which cocoa exerts these effects in humans. To do so, we studied the effect of cocoa consumption on the global 5mC levels of peripheral leukocyte DNA in a population with cardiovascular risk factors. Additionally, to identify the possible mechanism by which cocoa consumption influences DNA methylation, we investigated whether genetic variants of the enzymes involved in the provision of methyl groups (MTHFR and MTRR) and of the enzymes that catalyze the transfer of these methyl groups to DNA (DNMTs) affect the relationship between cocoa consumption and DNA methylation. Furthermore, we also evaluated *in vitro* the effect of a cocoa polyphenol extract treatment on the mRNA expression levels of these five genes related to the DNA methylation pathway (*DNMT1, DNMT3a, DNMT3b, MTHFR and MTRR*) using peripheral blood mononuclear cells (PBMCs) from humans.

## Materials and Methods

The protocols for this trial and supporting CONSORT checklist are available as supporting information; see Checklist S1, Protocol S1 and Protocol S2.

### Subjects

The population sample for the present study was selected from earlier studies [21], [22]. The population sample included: a) control group of 104 individuals from 254 participants [21]; b) cocoa product group of 110 participants [22]. The participants were recruited between April 2005 and June 2007. Inclusion criteria were community-dwelling men and women >20 years of age, with pre-hypertension (systolic BP: 120–139 mmHg or diastolic BP: 80–89 mmHG); stage 1 hypertension (systolic BP: 140–159 mmHg or diastolic BP: 90–99 mmHg); LDL-C ≥3.35 mmol/L [130 mg/dL] and ≤4.88 mmol/L [189 mg/dL]; and at least one other major CVD risk factor such as age >45 years in men and >55 in women, smoking habit, low HDL-C ≤1.03 mmol/L [40 mg/dL] in men and ≤1.19 mmol/L [46 mg/dL] in women, or family history of premature heart disease. Exclusion criteria included diabetes mellitus, any chronic disease, hypolipemic treatment, TG >3.97 mmol/L [350 mg/dL], BMI >35 Kg/m^2^, and a history of CVD. Participant eligibility or exclusion was assessed by the attending physician and was based of review of clinical records, followed by screening visits [21], [22].

### Study characteristics

For the cocoa group (45 men and 65 women), during a stabilization period of 2 weeks, all participants received the cocoa cream product (composition as described in **Table 1**) in order to assess the subject's tolerance to the product (13 g/unit; 1 g cocoa/unit, 6 units/d; 6 g cocoa/d; 465 Kcal/d) included in an isocaloric diet with 13% of total energy as saturated fatty acids (treated group) [22]. For the control group (46 men and 58 women), in the run-in period of 2 weeks duration, the participants consumed an isocaloric diet in which the percentage of saturated fatty acids in the diet was 13% of total energy [21].

**Table 1:** Nutritional composition of cocoa cream (13 g dose)*.

	Content per dose
Energy, Kcal	77
Carbohydrate, g	6.6
Protein, g	0.2
Total fat, g	6.0
Saturated fat, g	1.2
Stearic fatty acid, g	0.3
Monounsaturated fat, g	3.0
Polyunsaturated fat, g	1.4
n-6, g	3.0
Fiber, g	0.3
Vitamin E, mg	2.0
SFA/USFA	1∶3.5
MUFA/PUFA	1∶2.06
SFA/MUFA	1∶1.14
SFA/PUFA	1∶2.35

Kcal: kilocalories; g: gram; n-6: omega 6 (linoleic acid); SFA: saturated fatty acids; USFA: unsaturated fatty acids; MUFA: monounsaturated fatty acids; PUFA: polyunsaturated fatty acids.

* Nutrient composition calculated from data provided by the manufacturers and with USDA food composition tables.

Fasting blood samples from participants were obtained at the end of 2 weeks in each study for the isolation of peripheral leukocyte DNA.

All participants provided written informed consent prior to enrolment in the trial.

For the *in vitro* study, PBMCs were obtained from six adult volunteers (3 men and 3 women, aged 30–34 years) with their oral informed consent.

### Ethics statement

The study was approved by the Clinical Research Ethical Committee of the *Hospital Universitary Sant Joan de Reus* and at 3 Primary-Care Centers (Alcover, Vic, Centelles) where participants were recruited and the human experimentation was conducted. This study was conducted according to the guidelines established in the Declaration of Helsinki. The trial was registered with CinicalTrials.gov, numbers NCT00502047 and NCT00511420.

### Global DNA methylation detection

#### Genomic DNA extraction and digestion

The global methylation levels of peripheral leukocyte DNA from blood samples and cultured PBMCs were evaluated by measuring the 5mC content using reversed-phase HPLC. Blood samples were obtained from the treated and control subjects at week 2, and genomic DNA was isolated from the peripheral leukocytes using a cell package commercial kit (Servicios Hospitalarios, Spain). DNA was extracted from the PBMCs using a DNeasy® Blood & Tissue kit (Qiagen, S.G. Servicios Hospitalarios S.L., Barcelona, Spain). The DNA was quantified using a NanoDrop 1000 Spectrophotometer (ThermoFisher Scientific, Wilmington DE, USA).

DNA digestion was performed essentially as described elsewhere [23], [24] but with a few modifications. Briefly, 30 µl of genomic DNA was treated with RNase A (Sigma-Aldrich, St. Louis, MO, USA) and RNase T1 (Sigma) at final concentrations of 100 U/mL and 2000 U/mL, respectively, and incubated at 37°C for 2 h. The DNA was then extracted with phenol-chloroform, precipitated by sodium acetate (Sigma) and absolute ethanol, and washed twice with 70 per cent ethanol. Afterwards, the DNA was resuspended with 30 µl of 1× deoxyribonuclease I digestion buffer and 10 U of DNaseI (New England Biolabs, IZASA S.A., Barcelona, Spain), and the mixture was incubated overnight at 37°C. Sodium acetate (pH 5.2), zinc sulphate, and nuclease P1 (Sigma) were then added, and the mixture was incubated for an additional 7 h at 37°C. After the digestion, 1/10 volume of 10× alkaline phosphatase buffer (10 mM Tris-HCl, pH 8.0, 50 mM KCl, 1 mM MgCl_2_, 0.1 mM ZnCl_2_) and 10.8 U of alkaline phosphatase were added to the DNA solution. Additionally, 2 µl of tetrahydrouridine (Calbiochem, EMD Biosciences, La Jolla, CA, USA) was added to prevent the adventitious formation of dU by cytidine deaminase [25], and the solution was incubated overnight at 37°C. Finally, DNA hydrolysate was filtered using a 0.45-µm filter (Millipore, Carrigtwohill, Co. Cork, Ireland), and the samples were stored at −80°C until analysis.

#### Generation of Standard curves and HPLC analysis

Commercially available (TCI Europe, Borenveldgewes, Belgium) nucleotide standards for 2′-deoxyadenosine (dA; D0046), 2′-deoxyguanosine (dG; D0052), thymidine (dT; T0233), and 2′-deoxycytidine (dC; D3583) at 100 µM and 2′-deoxyuridine (dU) and 2′-deoxy-5-methylcytidine (5mC; D0046) at 5 µM were used to prepare a mixed sample of all six nucleotides to generate calibration curves for each sample batch. Linear calibration curves were obtained in the concentration range of 2.0 to 100.0 µM for dC, dG, dT and dA and in the range of 0.1–5.0 µM for 5mC and dU, spanning the expected concentration ranges of the samples. The daily precision was examined by analysing the deoxynucleoside standards (100 µM dC, dG, dT, and dA and 5 µM 5mC and dU) at the start and end of every experiment.

The 5mC content was measured using an Agilent 1100 Series liquid chromatograph (Agilent Technologies) equipped with a diode array detector (Agilent Technologies) at room temperature. The column was an Eclipse Plus C18 column (250×4.6 mm, 5 µm) with a ZORBAX Eclipse Plus-C18 guard column (12.5×4.6 mm, 5 µm) (Agilent Technologies). The mobile phase was 50 mM ammonium orthophosphate, pH 4.1, which was prepared by dissolving 50 mmol of diammonium orthophosphate in 50 mM orthophosphoric acid with a subsequent adjustment of the pH to 4.14. The flow rate was 1 mL/min, with a 20 µl injection volume, and the sample detection was set to 275 nm (UV).

For each sample, at least two separate aliquots of DNA were digested and chromatographed. All DNA samples were analyzed in duplicate, and we eliminated samples for which the difference in the 5mC content between duplicate samples was greater than 0.3%.

The area of each peak was used for the quantitative analysis. The sample concentrations were calculated from the curves using a linear regression, and the level of 5mC in the DNA samples was then expressed as a percentage of the level of dC, which was calculated using the following equation: %5mC = [5mC/(dC+5mC)]×100.

### Polymorphisms analysis

The analyzed SNPs were selected using two criteria. Because there are many SNPs in each of the three DNMTs, the majority of which are located in introns with unknown functional effects, we used HapMap (http://hapmap.ncbi.nlm.nih.gov/) to select haplotype tagging SNPs to include in the analysis (Tagger-Pairwise, Hap Map Data Phase III/Rel#2, Feb09, on NCBI B36 assembly, dbSNP b126 for the CEU population, r^2^ threshold ≥0.8 and MAF≥0.10). In addition, to select polymorphisms in genes that encode the MTHFR and MTRR enzymes, we used PubMed to identify SNPs that had previously been reported in the literature to have functional effects. To ensure a wide number of subjects who are homozygous for the rare alleles, we selected only those polymorphisms with a minor allele frequency in Europeans of at least 10%, as indicated by the SNP public database (dbSNP; http://www.ncbi.nlm.nih.gov/sites/entrez?db=snp). Finally, a total of two MTHFR (by rs number: rs180133, rs180131), two MTRR (rs1801394, rs1532268), three DNMT1 (rs2162560, rs759920, rs7253062), four DNMT3A (rs2304429, rs2289195, rs13002567, rs734693) and three DNMT3B (rs998382, rs4911263, rs2424932) SNPs were included.

Genotyping of the selected polymorphisms was performed using the Iplex Gold Sequenom technology (coordinacion.cegen@upf.edu).

### *In vitro* studies with PBMCs

#### Cocoa extract

The cocoa extract used for this study was produced from cocoa nibs. The total polyphenol content of this cocoa extract, measured using Folin-Ciocalteu's method, was 302.5 mg/g. The majority of the polyphenols were monomeric (catechin, 2.374 mg/g and epicatechin, 5.638 mg/g), dimeric (13.20 mg/g), trimeric (6.216 mg/g), tetrameric (1.571 mg/g) or oligomeric (5–9 units, 0.3 mg/g) proanthocyanidins (measured using ultraperformance liquid chromatography MS). The cocoa extract used in the *in vitro* experiments had similar characteristics to that used in the human intervention study.

#### PBMCs preparation and treatment

PBMCs were isolated from 20 mL EDTA anticoagulated blood of six volunteers by density gradient centrifugation using Ficoll-Paque™ PLUS (GE Healthcare Bio-Sciences AB, Uppsala, Sweden) and stored in liquid nitrogen. The cells were thawed by slow reconstitution with RPMI 1640 medium supplemented with 10% fetal bovine serum, 2% L-glutamine and 1% penicillin-streptomycin (Lonza, Barcelona, Spain). The PBMCs fraction was adjusted to 1.0×10^6^ cells/mL, and 1 mL of cells was aliquoted into each well of the 12-well flat-bottom plates.

To evaluate the effect of the cocoa extract on mRNA expression, the cells were treated with 10 µl of a cocoa extract with two final concentrations (25 mg/L and 50 mg/L) dissolved in 5% DMSO. The cells treated only with vehicle (5% DMSO) served as controls. The cells were incubated at 37°C in 5% CO_2_ for 3 h and 24 h. The cell culture experiments were performed in triplicate and repeated two times. After treatment, the cells were obtained by centrifugation at 700× g for 10 min at 4°C.

To evaluate the effect of the cocoa extract on the global DNA methylation, the PBMCs were treated with the highest concentration of cocoa extract (50 mg/L) for 72 h. The detection of the global DNA methylation in the PBMCs cultures was performed as described above.

#### Isolation of total RNA and quantitative real-time PCR

The total RNA from the PBMCs samples was extracted using a combination of Tripure Reagent (Roche Diagnostics, Barcelona, Spain) and an RNeasy® MiniElute™ Cleanup Kit (Qiagen, S.G. Servicios Hospitalarios S.L., Barcelona, Spain), dissolved in 22 µl of RNase free water and stored at −80°C. The RNA yield was quantified using a NanoDrop 1000 Spectrophotometer (ThermoFisher Scientific, Wilmington DE, USA).

The reverse transcription reactions were performed with 400 ng of RNA that was reverse transcribed to cDNA using the High-Capacity cDNA Reverse Transcription Kit (Applied Biosystems) following the manufacturer's protocol. An analysis of the relative gene expression for *DNMT1*, *DNMT3A*, *DNMT3B*, *MTHFR* and *MTRR* was performed using real-time quantitative PCR in a 7300 Real-Time PCR System (Applied Biosystems). For the amplification reactions, the TaqMan Gene Expression Master Mix and Inventoried Gene Assay Products (Applied Biosystems) containing 2 gene-specific primers and 1 TaqMan MGB probe (6-FAM dye-labelled) from Applied Biosystems were used (assay IDs DNMT1: Hs00154749_m1, DNMT3A: Hs01027166_m1, DNMT3B: Hs00171876_m1, MTHFR: Hs00195560_m1, MTRR: Hs00985015_m1). The thermal cycle conditions were as follows: first cycle at 50°C for 2 minutes followed by 95°C for 10 minutes, and 40 cycles at 95°C for 15 seconds followed by 60°C for 1 minute. A no-template control was included in each assay. *RPLP0* (Hs99999902_m1) was used as an endogenous control, and a vehicle control was used as a calibrator. The expression levels of the target transcripts in each sample were calculated using the comparative Ct method (2^−ΔCt^ formula) after normalization to *RPLP0* mRNA.

#### Assay of cell viability

The cocoa extract toxicity on cultured PBMCs was determined by measuring the amount of intracellular lactate dehydrogenase (LDH) in the culture supernatants. The PBMCs were incubated in 12-well plates at a density of 1×10^6^ cells/well with different concentrations of cocoa extract (1, 10, 50, 100, and 250 mg/L) for 24 h. After incubation, the LDH activity in the medium was measured by analyzing the NAD reduction using an LDH kit (QCA, Barcelona, Spain) according to the manufacturer's instructions and measuring the absorbance at 340 nm.

The LDH activity was expressed as the percentage of the total LDH cellular released following the addition of (0.1%) Triton X-100. The survival of the vehicle-treated control cultures receiving no cocoa extracts was defined as 100%, and that of the treated groups was expressed as a percentage of the control group. The results are given as the mean (± SD) of different incubations, each performed in triplicate.

### Statistical analysis

A deviation from the Hardy-Weinberg equilibrium for genotype frequencies at individual loci was assessed using standard chi-squared tests. To compare the means of continuous descriptive variables between treatment groups and to study the cocoa consumption response differences on the global DNA methylation between groups, an analysis of covariance was used (ANCOVA). The independent variables (covariates) were determined using a stepwise regression (successive steps). A Fisher exact test was used for the categorical variables. To study the cocoa consumption response differences on the global DNA methylation according to genotypes, ANCOVA was used for all of the analyses with the %5mC defined as the dependent variable and the treatment and covariates included in the model. The analyses were performed under the recessive, dominant, and codominant models. For the analyses of the mRNA expression levels and the global DNA methylation levels in PBMCs, a one-way ANOVA followed by a Tukey test or a two-way ANOVA was used.

With 104 and 110 subjects per group, the study has 90% power to detect a difference of at least 0.5% in %5mC assuming a SD of 0.800, with a global two-sided alpha of 5%.

The experimental results with continuous variables are expressed as the mean ± SEM or the mean ± SD, whereas the human data results of %5mC are expressed as the least-square mean ± SEM or the least-square mean and 95% confidence intervals (95% CI) for each genotype.

All statistical analyses were performed with SPSS version 17.0 software (SPSS Inc., Chicago, IL, USA). Statistical significance was considered at p≤0.05. For genotypes analysis, we applied step-down Sidak adjustment to correct for multiple testing and the two p values are presented.

## Results

### Global DNA methylation in human peripheral leukocyte DNA

We measured the global peripheral leukocyte DNA methylation content in a population that consumed 6 g/d of cocoa in the form of chocolate for two weeks (treated subjects) and in a population that did not consume any chocolate and thus served as controls. **Table 2** shows the characteristics of the participants of the study. No significant differences in age, gender, weight or body mass index were observed between the two groups.

**Table 2:** Characteristics of participants.

Variable	CONTROL	TREATED	P*
N	104	110	
Gender; male, N(%)	46 (44.2)	45 (40.9)	0.68
Age (years) (SD)	54.73±11.41	53.95±9.97	0.59
Weight (Kg) (SD)	72.06±10.78	74.07±11.09	0.19
Body Mass Index (Kg/m2) (SD)	27.33±3.36	28.05±3.26	0.12

SD, standard deviation.

* Differences in mean values were assessed by ANOVA adjusted by covariates.

**Figure 1:**
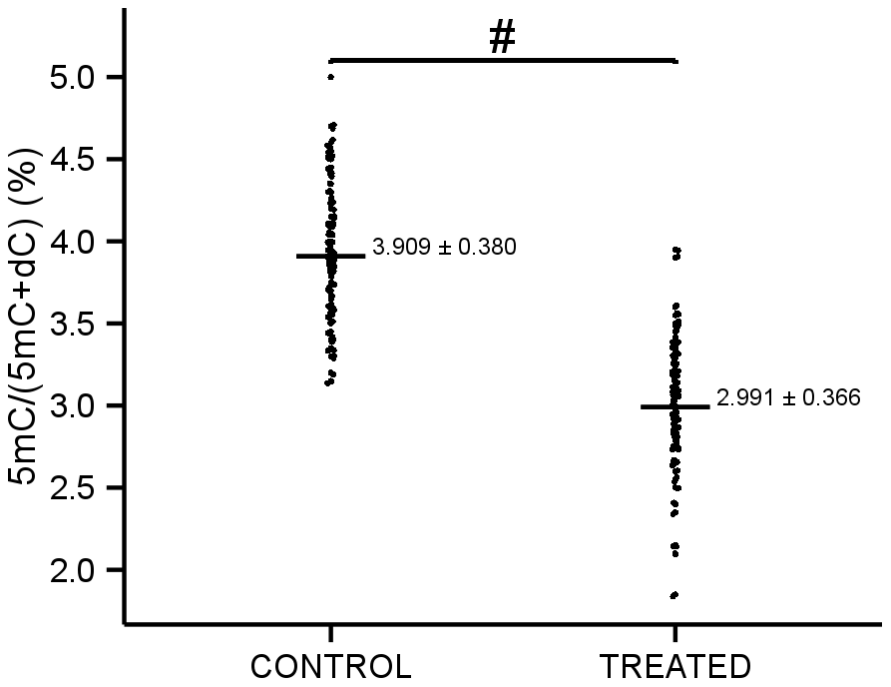
Global peripheral leukocyte DNA methylation levels in subjects. Global peripheral leukocyte DNA methylation values in subjects that consumed 6 g/d of cocoa (n = 110) and control subjects (n = 104). Methylcytosine levels (5mC), measured as percentage of total cytosine, were determined by HPLC. Relative to control subjects, mean±SD global peripheral DNA methylation levels were significantly decreased in treated subjects (ANCOVA). ^#^ p≤0.001.

We observed a statistically significant difference in the methylation status between the two groups analyzed. As shown in **Figure 1**, the %5mC of the peripheral leukocyte DNA was significantly lower in the treated subjects than in the control subjects (2.991±0.366 vs. 3.909±0.380, p<0.001). In our study, there were no statistically significant differences between men and woman in the treated or the control group, and no correlation was observed between the methylation value and the subject's age (data not shown).

Moreover, in the treated group, the %5mC levels were higher in the homozygote G subjects (mean (95% CI); 3.117 (2.991 to 3.242)) than in the carriers of the allele A (2.945 (2.869 to 3.021)) (p = 0.022) of the rs1801394 polymorphism in the *MTRR* gene, although the corrected p value was not statistically significant (p = 0.268). No association was observed between the others polymorphisms analyzed and the %5mC levels in total treated group. In stratified analysis by sex, we observed an association between the %5mC levels and two other polymorphisms in men treated group but none in women. Thus, the %5mC levels were lower in the homozygote A men (2.928 (2.786 to 3.069)) than in the carriers of the allele C (3.158 (2.996 to 3.321)) (p = 0.036) of the rs1801131 polymorphism in the *MTHFR* gene, and the %5mC levels were higher in the homozygote T men (3.143 (2.996 to 3.290)) than in the carriers of the allele C (2.900 (2.746 to 3.054)) (p = 0.026) of the rs998382 polymorphism in the *DNMT3B* gene, although the corrected p value were not statistically significant (p = 0.379 and p = 0.308, respectively). No association was observed between rs1801394, rs1801131 and rs998382 polymorphisms and the %5mC levels in the control group. Table S1 shows the global DNA methylation levels of peripheral leukocytes of participants (treated and control group) for each SNP analyzed.

### mRNA expression levels and global DNA methylation in PBMCs

Because the methylation values of the treated subjects were lower than those found in the controls, we addressed the mechanisms potentially causing the demethylation of DNA by comparing the expression levels of a set of genes related to the DNA methylation process in PBMCs. For this purpose, the PBMCs from six subjects were treated with a cocoa extract or with the vehicle for 3 h and 24 h. The composition of the cocoa extract used was similar to that used in the human intervention study. We assessed the transcription levels of the genes responsible for the DNA methylation (*DNMT1*, *DNMT3A* and *DNMT3B*) and of two other genes also involved in the DNA methylation process (*MTHFR* and *MTRR*).

When the PBMCs were incubated with cocoa extract for 24 h at a concentration in the range from 1 to 250 mg/L, the cocoa extract did not induce any decrease in cell viability at any concentration (data not shown). Thus, we chose the two lowest cocoa extract concentrations (25 and 50 mg/L) and performed the following experiments using these concentrations in PBMCs.

**Figure 2:**
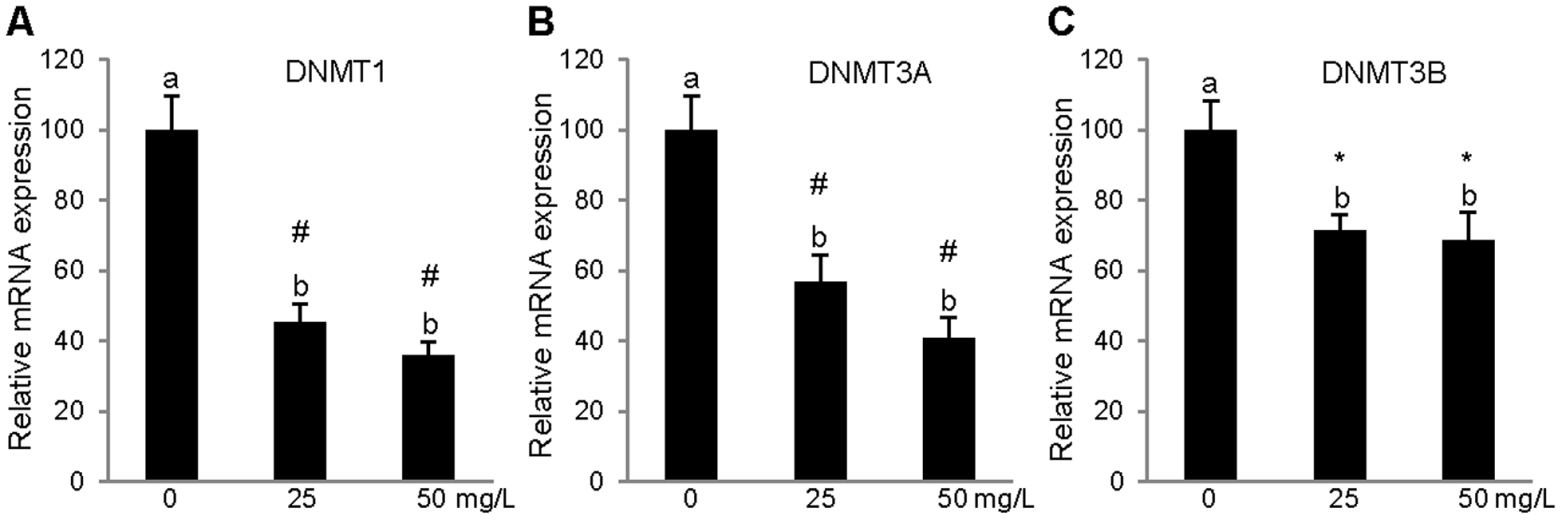
Relative gene expression levels of DNA methyltransferases in *in vitro* PBMC. Relative mRNA levels of DNA methyltransferases DNMT1 (a), DNMT3a (b), and DNMT3b (c) after 24 h treatment with cocoa extract (25 mg/L or 50 mg/L) or with vehicle. Results represent mean±SEM expression levels normalized to RPLP0. DNMT1, DNMT3a and DNMT3b expression were significantly reduced by cocoa treatment relative to control treated with vehicle alone (one-way ANOVA and Tukey's post hoc comparison). ^ab^Mean values not sharing the same letters were significantly different. *p≤0.05; #≤0.001.

**Figure 3:**
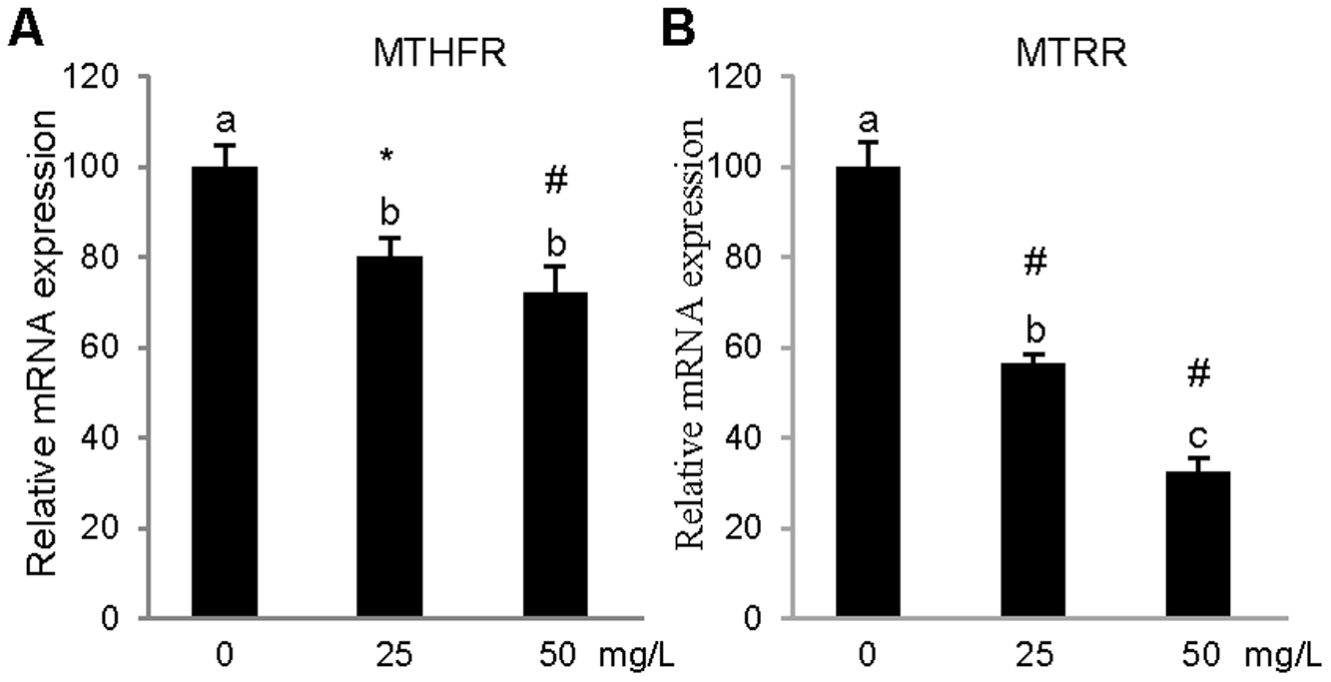
Relative gene expression levels of MTHFR and MTRR in *in vitro* PBMCs. Relative mRNA levels of MTHFR (a) and MTRR (b) after 24 h treatment with cocoa extract (25 mg/L or 50 mg/L) or with vehicle alone. Results represent mean±SEM expression levels normalized to RPLP0. MTHFR and MTRR expression were significantly reduced by cocoa treatment relative to control treated with vehicle alone (one-way ANOVA and Tukey's post hoc comparison). ^abc^Mean values not sharing the same letters were significantly different. *p≤0.05; #p≤0.001.

Our results indicated that the cocoa extract did not have any effect on the gene expression after 3 h of treatment (data not shown). Nevertheless, after 24 h, the cocoa extract had an effect on the mRNA expression of the genes studied in comparison with their respective non-treated controls in PBMCs. As shown in **Figure 2**, the two concentrations used (25 mg/L and 50 mg/L) significantly lowered the mRNA expression of the *DNMT1*, *DNMT3A*, *DNMT3B* genes, and the concentration that resulted in the maximal effect was 50 mg/L in PBMCs. Regarding the effect of cocoa extract on the *MTHFR* and *MTRR* gene expression levels, the PBMCs treated with both concentration of cocoa extract (25 mg/L and 50 mg/L) exhibited a significant repression of both genes compared with the PBMCs treated with the vehicle alone (**Figure 3**).

**Figure 4:**
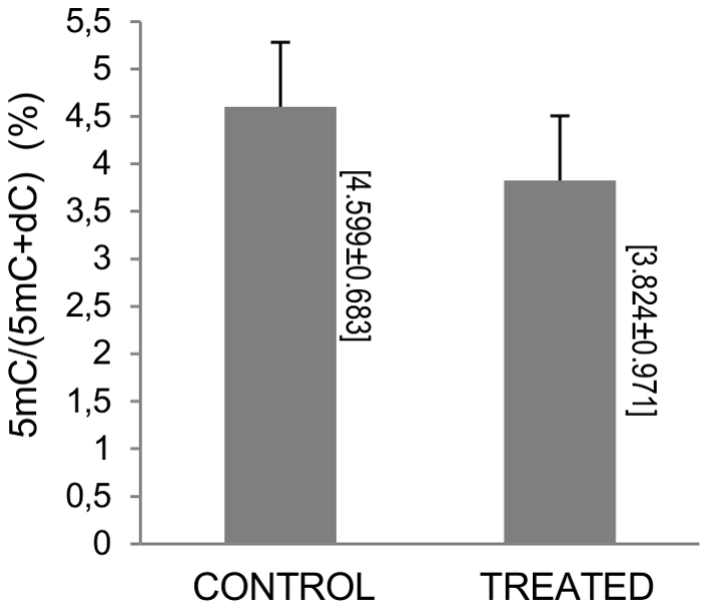
Global DNA methylation levels in *in vitro* PBMC. Global DNA methylation levels after 72 h treatment with cocoa extract (50 mg/L) or with vehicle. The treatment with the cocoa extract had a tendency to decrease the %5mC levels (p = 0.09). Results represent mean±SD.

We also determined the global DNA methylation in the PBMCs treated with the highest concentration of the cocoa extract (50 mg/L). The treatment with the cocoa extract had a tendency to decrease the %5mC levels (3.824±0.971 vs. 4.599±0.683, p = 0.09) (**Figure 4**).

## Discussion

The results of the present study demonstrate that cocoa consumption causes alterations in the global DNA methylation of peripheral leukocytes in humans with cardiovascular risk factors, lowering the %5mC levels. To our knowledge, this report is the first describing an epigenetic effect of cocoa that might be relevant for human health.

Unlike the genome, the epigenome is highly variable between cells and is dynamic and plastic in response to cellular stress and environmental cues. The role of the epigenome, specifically, the methylome has been increasingly highlighted and has been implicated in many cellular and developmental processes such as embryonic reprogramming, cellular differentiation, imprinting, X chromosome inactivation, genomic stability, and complex diseases such as cancer [26].

In this sense, the association between some cancer types and global DNA hypomethylation is well known [27], [28]. Nevertheless, studies have demonstrated that changes in the epigenome are not only common in cancer, but they are also involved in the pathogenesis of other diseases, including cardiovascular disease. Furthermore, results from various studies suggest that alterations in global DNA methylation of peripheral leukocytes are associated with the development of CVD [5], [6]. The population used in our study consists of subjects with cardiovascular risk factors, such as elevated LDL cholesterol and BP levels and low HDL cholesterol levels. In this sense, it has been shown an association between global DNA methylation in peripheral leukocytes and cardiovascular risk factors, including a positive association with total and LDL cholesterol [8], fasting glucose levels [8], hypertension [5], insulin resistance [7], and atherosclerosis [6], and a negative association with HDL cholesterol levels [8]. These risk factors are also associated with other important human diseases, such as obesity and diabetes. Thus, the consumption of food components that can modify these alterations, such as cocoa, may be beneficial for the prevention or treatment of diseases related to altered epigenetic mechanisms and an increase in the global peripheral DNA methylation levels, such as cardiovascular disease. However, more studies are needed to determine functional consequences of increased global DNA methylation levels in peripheral leukocytes for the development of human diseases.

Moreover, our results indicate that the effect of cocoa consumption on the global DNA methylation status of peripheral leukocytes depends on some genetic variants. Concretely, within the treated population in our study, the subjects with the MTRR A66G polymorphism and men with the MTHFR A1298C polymorphism had higher levels of global peripheral leukocyte DNA methylation compared with those without the MTRR or MTHFR polymorphisms. MTHFR and MTRR play central roles in the biotransformation of folate to form S-adenosylmethionine, the universal methyl donor in cells [3], [4]. Additionally, several studies have shown that these two polymorphisms are associated with reduced enzyme activity [29], [30], which may influence the pool of methyl-donor molecules and, therefore, DNA methylation [31], [32]. Our results suggest that cocoa consumption produces a more potent effect on the reduction of DNA methylation levels in humans with normal activities of MTHFR and MTRR enzymes. We also observed an association between cocoa consumption and the DNMT3B A38946G polymorphism on global peripheral leukocyte DNA methylation in men. This polymorphism is located in an intronic region of the *DNMT3B* gene and has never been studied previously. The results of our study suggest that this polymorphism may have a functional effect.

In the second part of the study, we tested the hypothesis that cocoa could decrease the global peripheral leukocyte DNA methylation via the regulation of key genes involved in this epigenetic process. Therefore, we performed *in vitro* experiments by treating PBMCs from subjects with a cocoa extract with its naturally occurring combination of polyphenols. In our study, low concentrations of polyphenols were sufficient to obtain a significant reduction in the mRNA levels of the *DNMTs*, *MTHFR* and *MTRR* genes. Interestingly, the reduction in gene expression was observed with each of the three main DNMTs analyzed, which is consistent with other reports that have shown that several dietary polyphenols, such as caffeic acid, chlorogenic acid, EGCG, catechin, epicatechin and genistein, are inhibitors of all three DNMTs [10], [13], [33]. Because the three DNMTs have been shown to exhibit some level of both maintenance and *de novo* methylation *in vitro* [34], our results in PBMCs suggest that the hypomethylation effect of cocoa could be produced, in part, through the down-regulation of these genes.

In our study, the ability of cocoa extract to decrease mRNA expression was correlated with its ability to decrease global DNA methylation in PBMCs, although the differences were not statistically significant. These findings reinforce our hypothesis and are compatible with the results observed in several studies in which the capacity of polyphenolic compounds to inhibit DNMTs was associated with alterations in DNA methylation *in vitro*. For instance, EGCG from green tea is known to lower DNMT activity, leading to decreased DNA methylation of several known TS genes, such as *p16^INK4a^*, retinoic acid receptor β, and methylguanine methyltransferase, in human oesophageal, prostate, breast, and oral cancer cells lines [10], [12], [35]. In addition to EGCG, other common dietary polyphenolic compounds, including catechin and epicatechin [10], genistein from soybeans [11], [33], caffeic acid and chlorogenic acid from coffee [13], and curcumin from curry [14], have also been shown to be demethylating agents, inhibiting the DNA methylation catalyzed by DNMTs of several TS genes or of the global genomic DNA [14] in different human cancer cells lines. Similarly, studies of a green tee polyphenol extract [36] and apple polyphenols [37] have provided analogous results. Moreover, in our study, the cocoa treatment of PBMCs also reduced the mRNA levels of the genes that encode the enzymes MTHFR and MTRR, which play an important role in the DNA methylation process [3], [4], confirming the effect of cocoa extract on DNA methylation.

Of particular importance is the fact that the *in vitro* results of this study corroborated the results obtained in the population study. Thus, whereas previous *in vitro* studies have shown that polyphenolic compounds have epigenetic effects that modulate DNA methylation, the data are weaker in animal studies. For instance, *in vitro* studies have shown that genistein induces DNA demethylation, but three animal studies have observed increased DNA methylation following the treatment [15]–[17]. In another study, feeding a composite of green tea polyphenols in drinking water to wild-type or transgenic adenocarcinoma of mouse prostate mice did not change either the global- or gene-specific DNA methylation status [18]. In our study, the cocoa dose consumed by the individuals (6 g/d) was consistent with the average human cocoa intake in countries with low consumption such as Spain [38], [39]. Additionally, the effective concentration of the cocoa extract for DNA methylation and mRNA expression modification was 50 mg/L. These concentrations are lower than those used in many other studies in cells lines, where the effective concentrations of polyphenolic compounds are higher than the plasma and tissue levels of these compounds generally observed after ingestion [12]. Moreover, it is also important to consider the fact that the biological activity of dietary polyphenols largely depends on the bioavailability of these substances. In this study, we have used a cocoa extract containing catechin, epicatechin, and dimeric and trimeric procyanidins as the major polyphenolic components. The bioavailability of cocoa polyphenols has been measured in several studies in humans, and monomeric flavonoids (catechin and epicatechin), dimeric and trimeric procyanidins have been detected in human plasma after cocoa consumption [20].

In conclusion, to the best of our knowledge, the present study is the first report to show that cocoa consumption has an effect on the global DNA methylation of peripheral leukocytes in humans with cardiovascular disease risk factors. Furthermore, our *in vitro* studies in PBMCs demonstrate that a cocoa extract has the ability to inhibit the expression levels of genes that are highly related to the DNA methylation process (*DNMTs*, *MTHFR* and *MTRR*). Nevertheless, further studies are necessary to evaluate the beneficial effects of global peripheral DNA methylation reduction by cocoa consumption.

## Supporting Information

Table S1 **DNA methylation levels for each SNP tested.**
(DOCX)

Checklist S1 **CONSORT checklist.**
(DOC)

Protocol S1 **Trial protocol.**
(PDF)

Protocol S2 **Trial protocol.**
(DOC)
