# Supplementary material for: Cocoa Consumption Alters the Global DNA Methylation of Peripheral Leukocytes in Humans with Cardiovascular Disease Risk Factors: A Randomized Controlled Trial
Source: PLoS One. 2013 Jun 26;8(6):e65744. doi: 10.1371/journal.pone.0065744 (PMC3694105; doi:10.1371/journal.pone.0065744)
Supplement: Table S1 — DNA methylation levels for each SNP tested. (DOCX) [file pone.0065744.s001.docx]

| **Gene** | **SNP** | **Genotype** | **% mC Adjusted mean [95% CI]** | |
| --- | --- | --- | --- | --- |
|  |  |  | **CONTROL** | **TREATED** |
| MTHFR | rs180133 | CC  CT  TT | 3.971 [3.854;4.088]  3.839 [3.736;3.942]  4.049 [3.852;4.246] | 3.011 [2.904;3.117]  2.952 [2.852;3.053]  3.044 [2.886;3.203] |
|  | rs180131 | AA  AC  CC | 3.899 [3.789;4.010]  3.909 [3.796;4.022]  4.006 [3.806;4.205] | 2.970 [2.879;3.061]  3.023 [2.916;3.130]  2.978 [2.734;3.223] |
| MTRR | rs1801394 | GG  AG  AA | 3.997 [3.860;4.133]  3.845 [3.739;3.950]  3.968 [3.821;4.116] | 3.117 [2.991;3.242]  2.963 [2.869;3.058]  2.913 [2.785;3.040] |
|  | rs1532268 | GG  AG  AA | 3.985 [3.868;4.102]  3.939 [3.837;4.041]  3.681 [3.496;3.865] | 2.971 [2.877;3.065]  2.998 [2.890;3.106]  3.053 [2.860;3.245] |
| DNMT1 | rs2162560 | GG  AG  AA | 3.921 [3.804;4.038]  3.878 [3.769;3.987]  3.939 [3.756;4.122] | 2.941 [2.827;3.054]  3.006 [2.914;3.099]  3.059 [2.881;3.237] |
|  | rs759920 | AA  AG  GG | 3.943 [3.817;4.068]  3.864 [3.755;3.973]  3.930 [3.772;4.088] | 2.923 [2.798;3.049]  3.013 [2.922;3.104]  3.029 [2.878;3.179] |
|  | rs7253062 | GG  AG  AA | 3.940 [3.825;4.054]  3.853 [3.745;3.961]  3.966 [3.778;4.154] | 2.939 [2.832;3.047]  3.013 [2.917;3.108]  3.059 [2.881;3.237] |
| DNMT3A | rs2304429 | AA  AG  GG | 3.859 [3.727;3.991]  3.945 [3.843;4.048]  3.867 [3.701;4.033] | 3.022 [2.910;3.133]  2.965 [2.869;3.061]  3.002 [2.834;3.170] |
|  | rs2289195 | GG  AG  AA | 3.901 [3.767;4.035]  3.872 [3.775;3.969]  4.020 [3.839;4.200] | 2.963 [2.844;3.082]  3.005 [2.912;3.097]  3.004 [2.840;3.167] |
|  | rs13002567 | TT  CT  CC | 3.916 [3.812;4.020]  3.907 [3.792;4.021]  3.836 [3.602;4.070] | 2.987 [2.891;3.082]  3.002 [2.896;3.108]  2.972 [2.772;3.172] |
|  | rs734693 | TT  CT  CC | 3.914 [3.811;4.018]  3.898 [3.793;4.003]  3.821 [3.324;4.319] | 3.016 [2.923;3.109]  2.959 [2.861;3.058]  3.040 [2.695;3.385] |
| DNMT3B | rs998382 | TT  CT  CC | 3.970 [3.870;4.070]  3.834 [3.712;3.957]  3.910 [3.691;4.130] | 3.044 [2.949;3.140]  2.951 [2.852;3.050]  2.884 [2.642;3.127] |
|  | rs4911263 | CC  CT  TT | 3.805 [3.682;3.928]  3.932 [3.836;4.027]  4.058 [3.852;4.264] | 2.979 [2.875;3.084]  2.983 [2.888;3.078]  3.078 [2.869;3.287] |
|  | rs2424932 | GG  AG  AA | 3.805 [3.650; 3.960]  3.897 [3.805;3.989]  4.045 [3.878;4.212] | 2.944 [2.820;3.068]  3.013 [2.926;3.101]  2.998 [2.820;3.176] |

**Table S1. Global DNA methylation levels of participants (control and treated group) for each SNP tested.**
