# Supplementary material for: Cocoa Consumption Alters the Global DNA Methylation of Peripheral Leukocytes in Humans with Cardiovascular Disease Risk Factors: A Randomized Controlled Trial
Source: PLoS One. 2013 Jun 26;8(6):e65744. doi: 10.1371/journal.pone.0065744 (PMC3694105; doi:10.1371/journal.pone.0065744)
Supplement: Protocol S1 — Trial protocol. (PDF) [file pone.0065744.s003.pdf]

Multicentre, comparative, double-blind, two-arm parallel clinical trial of the effects of treatment with Plantago ovata husk on the lipid profile of patients with hypercholesterolemia

Version no. 4, of May 20, 2005

**** Madaus, S.A. ****

1. SUMMARY

Type of application	Clinical trial with an active substance of a medicinal product in new conditions of use.
Name of the sponsor	Madaus, S. A.
Title of the clinical trial	Multicentre, comparative, double-blind, two-arm parallel clinical trial of the effects of treatment with *Plantago ovata* husk on the lipid profile of patients with hypercholesterolemia
Sponsor code	PLAN-EC-HIPERL-02
Principal investigator	Dr. Rosa Solà Alberich<br>Internal Medicine Service<br>Hospital Universitario San Joan<br>Reus (Tarragona)
Trial conduct site	Hospital Universitario San Joan<br>Reus (Tarragona)<br>Hospital St. Franciscus Gasthuis<br>Rotterdam (Holland)<br>Hospital Universitario Carlos Haya (Malaga)<br>Hospital San Vicente de Raspeig (Alicante)<br>CAP Alcover (Tarragona)<br>Centro de Salud Petrer I (Alicante)<br>Centro de Salud Petrer II (Alicante)<br>Hospital de la Pitié, Paris (France)
Authorising Independent Ethics Committee (IEC)	Independent Ethics Committees of the centres where the trial is performed<br>The reference IEC is the IEC of the Hospital Univ. San Joan de Reus (Tarragona)
Trial monitor	CRO Quintiles
Coordinator of the trial for the sponsor	Dr. Anna Anguera<br>Head of Investigation<br>Madaus, S.A.
Investigational Drug	Experimental: *Plantago ovata* husk (Plantaben)<br>Placebo: Microcrystalline cellulose
Trial phase	Phase IV-II
Objective of the trial	To determine the cholesterol-lowering effect of treatment with *Plantago ovata* husk in patients with moderate hypercholesterolemia in a low saturated fat diet

Disease studied	Patients with hypercholesterolemia, established as of LDL-C levels of ≥ 130 mg/dl
Primary endpoint	Reduction of LDL-C levels
Number of patients	290 patients
Duration of the treatment	The treatment will last 8 weeks, although it may be prolonged for a further 8 weeks depending on the response.
Duration of the study	Start: July 2005<br>Recruitment phase: 6 months<br>End: September 2006

2. CONTENTS

1. SUMMARY ... 1
2. CONTENTS ... 3
3. INFORMATION ... 5
4. RATIONALE AND OBJECTIVES ... 8
4.1. Rationale ... 8
4.2. Objectives ... 12
Primary endpoint ... 12
Secondary endpoint ... 12
5. TRIAL TYPE AND DESIGN ... 13
5.1. Development Phase ... 13
5.2. Detailed description of the randomisation process ... 13
5.3. Type of control and study design ... 13
5.4. Run-in periods ... 16
6. SELECTION OF STUDY POPULATION ... 16
6.1. Inclusion criteria ... 16
6.2. Exclusion criteria ... 16
6.3. Sample size ... 17
6.4. Interim analysis and interruption criteria ... 18
7. DESCRIPTION OF THE TREATMENT ... 19
7.1. Treatment regimens ... 19
7.2. Concomitant treatments ... 19
7.2.1. Permitted treatments ... 19
7.2.2 Prohibited treatments ... 20
7.3. Measures to evaluate compliance ... 20
8. TRIAL DEVELOPMENT AND ASSESSMENT OF RESPONSE ... 21
8.1. Endpoints ... 21
8.1.1. Primary endpoints: ... 21
8.1.2. Secondary endpoints: ... 21
8.2. Trial Development ... 22
8.3. Methodology ... 28
8.3.1. Laboratory analysis ... 28
8.3.2. Diet analysis ... 31
8.3.4. Treatment compliance ... 32
8.3.5. Collection of adverse events ... 32
9. ADVERSE EVENTS ... 33
9.1. General Information ... 33
9.2 Non-serious Adverse Events (NSAEs) ... 33
9.3 Serious Adverse Events (SAEs) ... 33
9.4. Notification of Adverse Events to the Health Authorities ... 35
9.5. Abnormal laboratory findings ... 37
9.6. Other Safety Considerations ... 37
9.7. Imputability criteria ... 38
10. ETHICAL CONSIDERATIONS ... 39
10.1. General considerations ... 39
10.2. Data Confidentiality ... 39
10.3. Trial Insurance ... 39
10.4. Independent Ethics Committee (IEC) ... 40
10.5. Informed consent ... 40
10.6. Confidentiality ... 41
10.7. Trial Budget ... 41
10.8. Insurance policy ... 41
11. PRACTICAL CONSIDERATIONS ... 42
11.1. Responsibilities of all the participants ... 42
11.1.1. Compliance with the protocol and amendments to the protocol ... 42

11.2. Control of compliance in the protocol ... 43
11.3. Data filing ... 43
11.3.1. Files and reports ... 43
11.3.2. File storage ... 44
11.4. Samples for clinical research and labels ... 44
11.5. Publication policy ... 45
12. STATISTICAL ANALYSIS ... 46
12.1. Descriptive analysis ... 46
12.2 Homogeneity of samples ... 46
12.3 Efficacy analysis ... 46
12.4. Analysis populations ... 49
12.4. Data management and Analysis centre ... 50
13. REFERENCES ... 51
14. SIGNATURE SHEET ... 53

ANNEX 1: Study evaluations diagram
ANNEX 2: Diet evaluations diagram
ANNEX 3: Patient Information Sheet
ANNEX 4: Informed Consent Form
ANNEX 5: Physical activity questionnaire
ANNEX 6: Food diary (Diet record)
ANNEX 7: 24h diet reminder
ANNEX 8: Sample collection, handling and storage procedure
ANNEX 9: Declaration of Helsinki
ANNEX 10: Public Liability Insurance
ANNEX 11: Standard Operating Procedures
ANNEX 13: Investigator's Brochure
ANNEX 14: Case Report Form
ANNEX 15: Economic Report
ANNEX 16: **Dietician's Manual**

3. INFORMATION

A. Identification of the Trial

Code: PLAN-EC-HIPERL-02
Version No. 3 of 1.09.2004

Title: Multicentre, comparative, double-blind, two-arm parallel clinical trial of the effects of treatment with *Plantago ovata* husk on the lipid profile of patients with hypercholesterolemia

B. Type of Clinical Trial

Clinical trial with a medicinal product, phase IV-II (exploratory-therapeutic)

C. Description of the investigational products

Investigational drug:

Generic name:	*Plantago ovata* husk
Presentation:	Sachets with 5 grams of granulate. Corresponds to 3.5 g of fibre.
Sample manufacturer:	Madaus S. A.
Trade name:	Plantaben

Placebo:

Microcrystalline cellulose + excipient

D. Sponsor of the trial

Madaus S. A.
C/ Foc 68 – 82
08038 Barcelona
Tel.: 93 – 298.82.00
Fax.: 93 – 431.98.85

E. Technical Director in charge of the manufacture of the samples

Dr. Jordi Briva
Technical Director
Madaus S. A.

F. Coordinator of the project for the sponsor and trial monitor

Coordinator for the sponsor:

Dr. Anna Anguera Vilà
Head of Investigation
Madaus S. A.
C/ Foc 68 – 82 08038 Barcelona

Tel.: 93 – 298.82.00
Fax.: 93 – 431.98.85
e-mail: aanguera@madaus.es

Trial monitor :

The trial will be monitored by the CRO Quintiles

G. Trial Coordinator

Dr. Rosa Solà Alberich
Internal Medicine Service
Hospital Universitario de San Juan
Reus (Tarragona)

H. Trial Centres and Investigators

Principal investigator:

Dr. Rosa Solà Alberich
Internal Medicine Service
Hospital Universitario de San Juan
Reus (Tarragona)

Investigators and participating centres:

Dr Manuel Castro Cabezas
Hosp. St. Franciscus Gasthuis
Rotterdam

Holland

Dr Mª Cruz Almaraz Almaraz
Hospital Universitario Carlos Haya
Málaga

Dr Xavier Luque
CAP Alcover
Tarragona

Dr José Vicente Vaquer
Centro de Salud Petrer I
Alicante

Dr. Domingo Orozco
Centro de Salud Petrer II
Alicante

Dr. John Chapman/ Dr Eric Bruckert
Hospital de la Pitié
Paris (France)

Dr. Luis de Teresa
Hospital San Vicente de Raspeig
Alicante

4. RATIONALE AND OBJECTIVES

4.1. Rationale

Cardiovascular diseases affect more than 30% of the population of the world's developed countries, and more specifically in Spain they account for more than 36% of deaths.[1]

One novel aspect of the therapeutic programme is the importance being acquired by non-pharmacological instruments, including diet and physical activity, with a view to reducing the risk of presenting coronary and cardiovascular diseases.[2,3] This new approach, called therapeutic life-style changes,[2] has two purposes, on the one hand the prevention and treatment of the high levels of low density lipoprotein cholesterol (LDL-C)[2] in plasma, the greatest risk factor in cardiovascular diseases. On the other hand, these measures target the reduction of the presentation of the actual cardiovascular events.

For all these reasons, diet-based therapy is the cornerstone of strategies for lowering plasma LDL-C and for reducing the risk of cardiovascular disease. Nevertheless, the optimal diet for protecting against cardiovascular diseases is a question of research and debate.

For many years, care has focused on the intake of fat and the replacement of saturated fatty acids by mono- and polyunsaturated fatty acids. Subsequently, the intake of carbohydrates has been well studied, and part of the interest lies in the relationship between different fibre-rich foods and in whether the type of fibre (soluble or insoluble) or the different types of fibre provided by foods (wholemeal cereals, fruits, greens and vegetables) can have a protective cardiovascular effect.

Ascertaining the quantity of fibre present in the food presents methodological difficulties, and in most cases the data used come from food composition tables. In our medium, the fibre content of the majority of foods of vegetable origin[4,5] was determined directly, and the intake is estimated at about 828 g daily[6] (about 576 g of greens, vegetables and fruit; 233 g of cereals, 312 g of legumes and 8 g of dried fruits). The quantity of total fibre is 18.5 g and soluble fibre 6.5 g a day[5]. These quantities are typical of a Mediterranean country and express better consumption conditions, since in practice and in our environment only 20% of the population eats these quantities[7,8].

What are the key elements of the diet in reducing levels of LDL-C and cardiovascular diseases?

At the moment, strategies focus on reducing the content in fatty acids to less than 7% of total energy and less than 200 mg daily of cholesterol.[2] Furthermore, the intake of *trans*-type fatty acids should be low, less than 2% of total energy.[3]

These cholesterol-lowering measures can be optimised through the introduction of viscous (soluble) fibre and vegetable steroids or their saturated (stanol esters) form.[2]

What are the current recommendations on fibre intake to reduce cholesterol?

In the United States, values have been dictated for total food fibre (Dietary Reference Intake, DRI) for the first time ever and generally, and are between 38 and 25 g a day, on the basis of 14 g of fibre per 1000 Kcal. The Adult Treatment Panel (ATPIII)[2] proposes an intake of 10 to 15 g of viscous (soluble) fibre to reduce plasma LDL-C.

The addition of soluble fibre provided by food and by the psyllium, pectin, oats or guar supplements can have an additional cholesterol-lowering effect over and above that which is attained with the reduction of saturated fatty acids (<7% of total energy) and the reduction of the intake of cholesterol (<200 mg/day).

In Europe and Spain, the cardioprotective diet is addressed in the context of a healthy life-style, although the recommended quantity of fibre is not specified.

Do foods cover total and soluble-type fibre needs?

If the data on vegetable origin food of our environment are taken into account, and in the optimal, albeit minority situation, in the best of cases the daily intake of total fibre is 18 g and that of soluble fibre is 6 g.

If the recommended quantities of viscous (soluble) fibre are between 10 and 25 g daily, only 6 g a day are provided with a high intake of vegetable-origin food from the diet. Thus, soluble fibre supplements play an important role in reaching the recommended quantities. One factor that should be taken into account is the viscosity of soluble fibre, as it seems to be the determining factor in the mechanisms of action on the metabolism of cholesterol.[9]

Efficacy

What is the effect of soluble fibre on LDL-C?

The effect of the intake of 10 g of soluble type dietary fibre in the context of a low saturated fat diet produces a reduction of 5% in plasma LDL-C.[2]

Generally speaking, the intake of 10 g of soluble fibre daily is thought to reduce LDL-C by 5%. This effect can also be assessed as 10 g of soluble fibre producing a reduction of 7.5 mg/dl of LDL-C.[10,11,12]

What does the individual response to soluble fibre depend on?

Each one of the metabolic steps involved (absorption, transport, storage) is regulated by a large number of genes that interact with each other and with the environment. These genes present relatively common variations in the general population, called polymorphisms. There is one for every 200 nucleotides, about 10 million in the genome, of which 250 in 15 genes are related to the lipid metabolism.

Despite high genetic variability, there is a number of genes which by virtue of their functional importance or the prevalence of relevant mutations in the population, may be regarded as very determinant genes.

More specifically, the individual response to food changes may be explained by some of the polymorphisms involved in the metabolism of LDL or HDL. More specifically, the response of LDL-C may be related to the polymorphisms of apolipoprotein E, the levels of high-density lipoprotein cholesterol (HDL-C) to the polymorphisms of the cholesterol ester transfer protein (CETP), and triglyceride plasma concentrations to the polymorphisms of apoliprotein A-V. The intake of soluble fibre is subject to individual factors and it is unknown whether there is a specific polymorphism that intervenes in fibre mechanisms on lipid metabolism , although on the basis of our previous results, the polymorphisms of the Fatty Acid Binding Protein (FABP type 2) at intestinal level in charge of the absorption of fatty acids could be candidates.[13]

The fact that viscous (soluble) fibre increases the losses of bile acids at intestinal level[9] renders it necessary to look for specific genes at this level of metabolism.

Safety

What are the adverse effects of the intake of soluble fibre?

Alterations of bowel movement such as flatulence and abdominal distension may present with the intake of soluble fibre at the beginning of treatment, but will disappear after a few days of follow-up.

The interaction between soluble fibre and the absorption of liposoluble vitamins and minerals must be recorded, although in most studies no changes have been detected between the group of patients taking soluble fibre and placebo consumers.

What is the aim of this study?

The effect on plasma lipids of the soluble fibre obtained from the husk of *Plantago ovata* seeds is somewhat unknown, although some studies using this type of fibre are beginning to be published. This fibre is marketed in Spain as a medicinal product and its extension to the rest of Europe is being addressed.

If soluble (viscous) fibre can optimise the effects of the cholesterol-lowering diet (reduction of the content of saturated fatty acids and cholesterol), supplementing this type of fibre takes on special relevance.

Our interest lies in ascertaining the cholesterol-lowering effect of a specific type of soluble fibre present in the husk of *Plantago ovata* seeds.

The intake of a high quantity of fibre-rich vegetable origin products provides about 6 g of soluble fibre a day.[6] In most cases, besides boosting the intake of this type of food,[3,14,15] treatment with a *Plantago ovata* husk product contributes to achieving the recommended 10 to 25 g of soluble fibre in the diet, thus controlling the lipid profile.[2]

Reality tells us that one part of the asymptomatic population presents a high cardiovascular risk due to the accumulation of several risk factors[17]: gender, age, smoking, systolic blood pressure and cholesterol or total cholesterol/HDL cholesterol. A large part of the Spanish population are smokers (63%),[1] another important percentage of the citizens have high blood pressure (higher than 40% [blood pressure ≥140 mm Hg or >90 mm Hg or in treatment])[1] and half our population presents high blood cholesterol values (more than 200 mg/dl).

High blood pressure can also be modified by fibre, and its effect on blood pressure seems moderate and independent.[18] However, there are no data on the action of *Plantago ovata* husk on the reduction of blood pressure levels.

In clinical practice, in order to reduce cardiovascular risk,[3,14] aside from the diet, many patients require treatment with cholesterol-lowering drugs, mainly statins.[3,1] For this reason, it will be interesting to provide data on the effects of the statins in combination with the administration of soluble fibre. This approach is based on the different modes of action of both: whereas viscous fibre increases the loss of bile salts through the intestine, the statins, by means of the inhibition of 3-hydroxy-3-methylglutaryl coenzyme A reductase, reduce the synthesis of cholesterol. Nevertheless, it has yet to be demonstrated whether the combination of a soluble fibre- and statin-based drug provokes a synergistic additive effect, or whether, on the other hand, its cholesterol-lowering effects are neutralised. This study uses simvastatin,[16] a regular and low-cost statin, which has a proven cholesterol-lowering additive effect with a fibre-rich diet.[16] Simvastatin at a dose of 20 mg a day provides a 20-30% reduction in LDL-C.

Treatment with statins will be initiated in patients who fail to reach desirable concentrations of 130 mg/dl of LDL-C[3,14] with soluble fibre, and it will thus be possible to study the effects of the combination of soluble fibre and statins.

Hypothesis

Soluble fibre may contribute to a reduction of LDL-C, and the combined effect with a statin (20 mg of simvastatin) may achieve an optimisation of the cholesterol-lowering effects in adults with several cardiovascular risk factors.

4.2. Objectives

Primary endpoint

To determine the capacity to *reduce by 5% the concentrations of plasma LDL-C* by treatment with soluble fibre *–Plantago ovata* husk– added to a low saturated fat diet in patients with moderate hypercholesterolemia.

Secondary endpoint

- To study the combined cholesterol-lowering effect of treatment with *Plantago ovata* husk and statins to achieve the therapeutic objective.
- To analyse the effect of *Plantago ovata* husk fibre on blood pressure.
- To study the effect of the combination of statins with *Plantago ovata* husk on blood pressure.
- To evaluate whether the response on plasma lipids is modulated by the polymorphisms of apoliprotein E, cholesterol ester transfer protein (CETP), apolipoprotein A-V and FABP 2.
- Furthermore, possible variations of the effects of the soluble fibre and the statins according to the characteristics at the beginning of the study, LDL-C level, gender or BMI will be studied.

5. TRIAL TYPE AND DESIGN

5.1. Development Phase

Phase IV-II study

5.2. Detailed description of the randomisation process

The eligible subjects will be randomly assigned to treatment A or B in a ratio of 1:1. With the aim of minimising potential bias in the result of this study because of the subjects, investigators or study personnel, in relation to the safety, efficacy or well-being of the study products, the study design will be double-blind. The subjects will be randomly assigned to treatment in a ratio of 1:1; the probability of any subject receiving either of the two treatments described for this study will therefore be 0.5. The investigator or the study personnel will include subjects suitable to participate in the study sequentially by number of subject and centre, starting with the first patient and centre number of the numerical series assigned by MADAUS. The sponsor responsible for the randomisation will guarantee the randomisation code. The authorised personnel will provide the randomisation code. The randomisation list consists of 09 centres, each centre containing 25 blocks of 4 patients per block; the final list being of 999 patients. This list has been programmed by Quintiles Trans. Version 8.2 of the SAS statistical package has been used.

5.3. Type of control and study design

This study is a phase IV-II study.
The study design will be multicentre, comparative, randomised, double-blind and parallel, with two arms. It is designed to evaluate, in the first part, the separate effects of *Plantago ovata* husk, and in cases where the therapeutic objective is not attained, the combined action of this fibre and a statin (20 mg of simvastatin) in adults with several cardiovascular risk factors will be studied (see figure 1).

Figure 1. ***Plantago ovata*** **Study design**

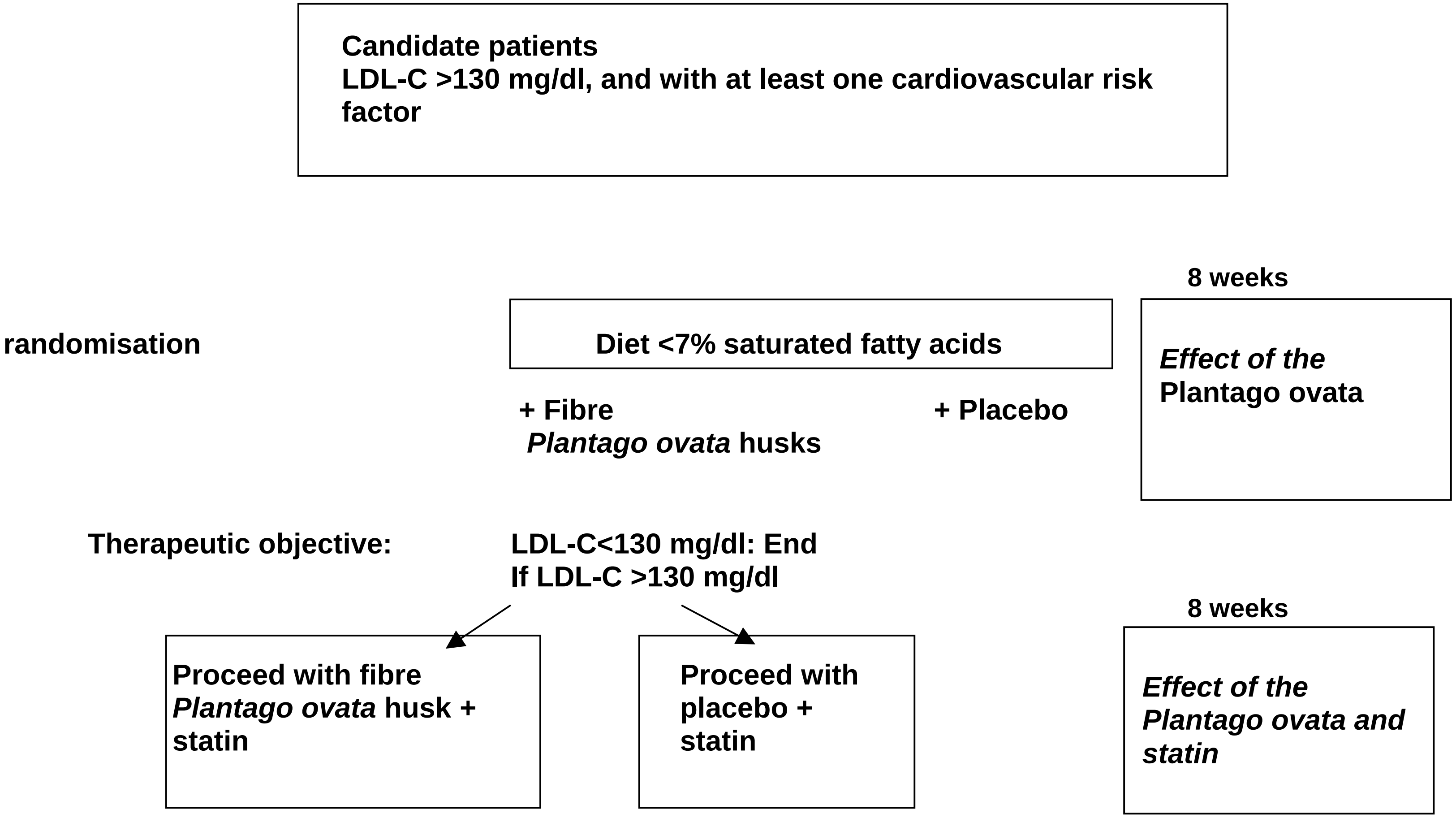

5.4. Run-in periods

The study includes an initial run-in period S-2(D-15) in which the patients will be given an appointment to ascertain whether they comply with the inclusion/exclusion criteria, they will be informed about the study characteristics and will also be asked to provide two blood samples, to be taken on days D-10 (± 2) and D-7 (± 2). Mainly, these two analyses are aimed at determining the patients' baseline LDL-C value. If it is within the margins established as an inclusion criterion, the patient will be informed of this at visit S0 and will be randomised.

6. SELECTION OF STUDY POPULATION

All men and women aged above 20 years, with an LDL cholesterol higher than or equal to 130 mg/dl and lower than or equal to 189 mg/dl will be invited to participate in the study. These patients must have at least 1 cardiovascular risk factor.

The following are regarded as risk factors:
* Age above 45 years in men and above 55 years in women,
* Smoker
* High blood pressure
* HDL-C ≤40 mg/dl in men and ≤46 mg/dl in women
* Family background of early heart disease

6.1. Inclusion criteria

1. Levels of LDL-C between >130 mg/dl and <189 mg/dl
2. Present at least 1 of the cardiovascular risk factor defined in the preceding section.
3. Be capable of following diet guidelines
4. Patients who have given informed consent.

6.2. Exclusion criteria

1. Triglycerides >350 mg/dl (threshold that allows to determine LDL-C according to the Friedewald’s formula).
2. Chronic alcoholism.
3. BMI>35 kg/m^2
4. History of cardiovascular disease.
5. Treatment with statins prior to the beginning of the trial and who have not dropped out at least 2 months before the beginning of the study.
6. Have diabetes mellitus (at least 2 glycemias, in fasting, above 126 mg/dl).
7. Renal disease (creatinine plasma levels > 124 μmol/l (1.4 mg/dl) for women and > 133μmol/l (1.5 mg/dl) for men.
8. Have acute infectious diseases, neoplasms, severe liver failure, chronic respiratory failure or associated endocrine diseases.

9. Other conditions, such as special nutritional requirements or medications that may affect lipid metabolism or blood pressure.
10. Participate or have participated in a clinical trial in the last 3 months.
11. Incapacity to follow the study.
12. Allergy to *Plantago ovata.*
13. History of gastrointestinal disease that may alter the absorption of nutrients.
14. Intestinal obstruction or pseudo obstruction.
15. Depression syndrome or self-injuring ideation.

6.3. Sample size

Total sample: 290 patients (145 per group)
The results of an earlier study by Sprecher 1993, measuring the effect of 10.2 g of *Plantago ovata* a day in the reduction of the levels of LDL cholesterol were used to calculate the sample.

The value of the effect to be detected (d = 7.5 mg/dl) is equivalent to a reduction of 5% of LDL in patients with LDL baseline values of 150 mg/dl.

The working hypothesis is:

H_0: Δ LDL *plantago ovata* - Δ LDL placebo < $|7.5|$ mg/dl

H_1: Δ LDL *plantago ovata* - Δ LDL placebo $\geq$ $|7.5|$ mg/dl

Where Δ LDL = final LDL – baseline LDL

For a statistical significance of α = 0.05 and a power of 80%, with a standard deviation of 20 mg/dl in the ΔLDL, the sample size required to detect significant differences of 7.5 mg/dl between the group treated with *Plantago ovata* and the group treated with placebo is 112 patients per group, for which, adjusted to an expected percentage of losses of 15%, a total of **270 patients** will be required.

After treatment, approximately 75% of the patients treated with soluble fibre (84 patients) and 85% of those treated with placebo (95) will not have 130 mg/dl of LDL-C in plasma. A statin will be added to all these patients' treatment.

It is assumed that the combination of soluble fibre with statins will cause the mean value of LDL-C to fall from 157 mg/dl to 125.6 mg/dl (ΔLDL soluble fibre + statin = 31.4 mg/dl). In the placebo + statin group from 161 mg/dl to 136.85 mg/dl (ΔLDL placebo+statin = 24.15 mg/dl).

For a statistical significance of (α=0.05), a power of 80%, a standard deviation in the ΔLDL of 18.5 mg/dL and a ratio of 0.85 between groups treated with placebo for 0.75 treated with soluble fibre, **the sample size** necessary to detect differences of

7.25 mg/dl in the ΔLDL between the group treated with statin+soluble fibre and the group treated with statin + placebo is 111 patients in the placebo group and 98 patients in the soluble fibre group.
As the percentage of failure in the objective of LDL-C equal to or lower than 130 mg/dl is 75% in the patients treated with soluble fibre and 85% in those treated with placebo, adding a 10% of patients lost to follow-up means that a **total sample of 290 patients (145 per group)** will be required.

6.4. Interim analysis and interruption criteria

The study withdrawal criteria will be:

- The presence of serious adverse events that lead patients to stop taking the product.
- The appearance of a serious disease with hospitalisation criteria.
- The taking of drugs other than those described as permitted.
- Non-compliance with the diet, defined by the following criteria:
  - energy provided by lipids more than 40%.
  - energy provided by saturated fatty acids higher than 15%.
- Non-compliance with fibre treatment. Compliance below 80% will be grounds for withdrawal.

6.5. Approximate duration of the recruitment period depending on the number of patients available

The recruitment period will have an approximate duration of 6 months.

For reasons of logistics, each participating centre should include a total of between 25 – 60 patients.

7. DESCRIPTION OF THE TREATMENT

7.1. Treatment regimens

During the initial treatment period (8 weeks) the patients will be given either the treatment with *Plantago ovata* husk (Plantaben) or placebo, according to the randomisation group.

The dose will be 20 g p.o. daily in the form of effervescent powder. This is the usual dose of the product in clinical practice.

The product will be distributed into 4 doses of 5 g, taken at main meal times, taking one dose at breakfast time, another at lunchtime and two with the evening meal.

The dose of fibre contained in Plantaben will total 14 g/d.

The duration of the treatment with each product will be 8 weeks. Subsequently, patients that do not fulfil the therapeutic objective will be given an added statin for a further 8 weeks. Patients who reach the therapeutic objective in the first 8 weeks will end follow-up at this time.

The statin chosen is Simvastatin 20 mg/day.

The fibre will be given at least 15 minutes before breakfast, lunch and the evening meal. The contents of the sachet should be dissolved in 200 – 250 ml of water and taken immediately.

The Simvastatin will be taken at night, 2 hours after the evening meal.

If the patient is regularly taking another medication, the time he/she takes it should be checked in order to avoid interference with the taking of the fibre and the statin or both.

If the regular medication is taken in the morning, it will be taken 2 h after breakfast to simulate the situation of fasting. In all cases, the medication should be taken 2 h after the meal.

7.2. Concomitant treatments

7.2.1. Permitted treatments

. Diuretics
. Beta-blockers
. Calcium channel inhibitors
. Angiotensin-converting enzyme inhibitors (ACEI).
. Angiotensin II receptor antagonists (AIIRA).

7.2.2 Prohibited treatments

- Statins (during the first 8-week period)
- Resins
- Fibrates
- Nicotinic acid and derivatives
- Ezetimibe
- Anticoagulants
- Minerals and vitamins
- Digoxin

It should be emphasised that patients may have followed some of the prohibited treatments for up to a minimum period of two months before their inclusion in the study.

All the drugs that the volunteer may take must be recorded.

7.3. Measures to evaluate compliance

Fibre phase

Each participant will be furnished with the fibre treatment, packaged in sachets. Delivery will be at the beginning of the intervention phase (S0) and at the visit after one month (S4). Similarly, at this visit (S4) the participant will return the containers of medication issued at the previous visit (used and unused) and the sachets taken will be counted. If low intake is detected the participant should be warned.
The participant will be given the treatment for the following four weeks at visit S4. At the end of this period (S8), all the containers of medication (used and unused) should be returned to check the sachets taken.

Fibre + statin phase

Patients who have to continue with the treatment will be given new medication for 4 weeks, with the statin added. At the week S12 visit each participant will return the medication corresponding to the fibre/placebo and the statin (used and unused) and both will be counted (fibre and statin). If low consumption is detected, the participant should be warned.
The participant will be given the treatment for the following four weeks at this same visit (S12). At the end of the period (S16), all the medication containers (used and unused) will be returned and the number of sachets and tablets taken will be confirmed.

A treatment compliance of higher than 80% will be regarded as suitable.

In view of the importance of diet compliance by the participants throughout the study, a dietician in each centre will be dedicated to the study from the preparation phase.

In order to evaluate diet compliance and detect possible deviations from the defined recommendations, the following procedures will be implemented:

- diet records for 3 days, which will be delivered at the medical visits. (Annex 6)
- Similarly, 24-h diet reminders will be made by phone without notice to guarantee compliance. (Annex 7).
- In order to achieve maximum precision in the calculation of food intake, the volunteers will receive training on how to make measurements at home during the first interview with the dietician. At the following visits by the volunteer to the dietician, the way the usual measures are assessed will be checked.

8. TRIAL DEVELOPMENT AND ASSESSMENT OF RESPONSE

8.1. Endpoints

8.1.1. Primary endpoints:

The primary endpoint is the reduction of LDL-C, evaluated by the mean of two determinations obtained at the end of the therapeutic intervention with soluble fibre and statins.

8.1.2. Secondary endpoints:

The secondary endpoints are:

The effect will be analysed of the soluble fibre and consuming soluble fibre and a statin on:

- *The total cholesterol, the triglycerides, the HDL cholesterol, the apolipoproteins (apo) B-100 and A-1 and the lipoprotein (a).*
- *The oxidation markers: alpha tocopherol and gamma tocopherol in LDL, resistance of LDL to oxidation (lag time), LDL oxidation “in vivo” and lipid lipoperoxides.*
- *The inflammation markers: IL-1beta, interleukin-6, TNF-alpha, amyloid-A, high sensitivity C-reactive protein, fibrinogen, leukocytes.*
- The indicators of antithrombotic activity: folic acid, homocysteine, plasminogen activator inhibitor (PAI-1).
- The individual response to the plasma lipids and their modulation by apoliprotein E polymorphisms, cholesterol ester transport protein (CETP), apolipoprotein A-V, FABP 2, ABCG5 and ABCG8, fibrinogen, fibrinogen activator inhibitor, angiotensin converting enzyme and other polymorphisms which might describe their involvement in the development of cardiovascular diseases.

- Fasting complement component 3 (C3)

- Mannose-binding lectin (MBL) activity
- Remnant-like particle cholesterol concentration (RLP-C)
- Parameters of cholesterol absorption and synthesis: plant sterols (campesterol and beta-sitosterol, reflecting cholesterol absorption) and lathosterol (reflecting cholesterol synthesis)
  - Determination of atherogenic apo B-containing particles (VLDL, IDL and LDL subclasses),
- HDL particle phenotype and HDL particle composition
- Cholesterol ester transfer protein activity

8.2. Trial Development

See Annex 1 and 2

The study design is described schematically below:

- Inclusion period: Week –2 (D–15) , D-10 and D-7 (D-5)
- Randomisation and initiation of the intervention: Week 0 (S0).
- End of the 1st intervention period: Week 8 (S8).
- Beginning of 2nd intervention period (fibre + statin): from week 8 (S8)
- End of the 2nd intervention period: Week 16 (S16)

Visit schedule:

Visit S -2 (Day -15)

The visits will include several aspects:

- ➢ Clinical
- ➢ Analytical
- ➢ Dietary

➢ Clinical

Contact will have been made with all the possible participants from the centres for the systematic control of people in whom hypercholesterolemia has been detected. Similarly, patients from the lipid clinic of each centre will have been contacted.

Contact will be by a letter and/or by telephone call explaining the detailed contents on the **Patient Information Sheet.** (Annex 3)

Patients who agree to participate in the study will be invited to this initial visit to confirm that they meet the selection criteria. They will be given the patient information sheet and they will be invited to sign the informed consent form (Annex 4) for their participation in the trial.

At this visit a detailed clinical history will be made, particularly with regard to:

- Age
- Smoking
- Intake of alcohol
- Blood pressure
- Background of cardiovascular disease
- Concomitant diseases: diabetes mellitus, renal failure, serious acute infectious diseases, neoplasm, severe liver failure, chronic respiratory failure or associated endocrine diseases.
- Concomitant pharmacological treatments.
- Physical activity.
- The patient's diet

The physical activity of each participant will be assessed according to a specific questionnaire (Annex 5).

The physical examination will include the determination of blood pressure, weight, height, BMI and waist size.

The participant's weight will be determined: on one occasion the patient will get off the scales and will then be reweighed; the mean value of the 2 weighings will be calculated.

The height of the patient will be measured in the following way: the subject will breathe in and the height meter will be lowered to determine height. The measurement will be repeated and the mean value of the 2 measurements will be calculated.

Patients that comply with the selection criteria will be given the Patient Information Sheet and will be asked to **sign the Informed Consent Form** (Annex 4) to participate in the trial.

Analytical

The patient will be given the blood test request form for extractions on days D –10 (±2) and D –7 (±2). The mean lipid profile will be calculated from these two blood tests and will be used as the baseline LDL-C value. The results should be available to the investigator at visit S0 to confirm that the patient complies with the analytical inclusion criteria. The analyses on the D-7 will be taken as the baseline for general haematological and biochemistry parameters. The genetic polymorphisms of this sample will also be analysed.

Dietary

Each patient will be given a diet record for 3 days, two working days and one holiday, which they will complete and return at visit D-7. The patients will be instructed on how

to complete the record as precisely as possible, as indicated in the methodology section.

The patients will return the diet record on the day of the second analysis, i.e. D-7 (±2).

The dietician will have 7 – 10 days to analyse the data in the diet record and calculate patient energy requirements. He or she will thus be able to design the isocaloric diets for each case, which will be given to the patients at visit S0.

Visit S0

This visit will be made no later than 9 days (7±2) as of the date of the last blood sample extraction.

The visit will include several aspects:

- Clinical
- Analytical
- Dietary

- *Medication*

- *Clinical*

A clinical history similar to that of S-2 will be made to confirm compliance with the inclusion criteria

Blood pressure and weight and height will be measured to calculate BMI at the physical examination.

- *Analytical*

The results of the mean LDL-C value obtained in the two analyses made on D–10 and D–7 will be noted. Similarly, the values obtained in the analysis on D-7 will be noted as baseline biochemistry and haematological parameters.

Furthermore, each patient's genetic polymorphism results will be collected at this visit.

- *Dietary*

On the basis of the detailed analysis of the record returned by the patient on D-7 and the calculation of the energy needs (according to the Harris-Benedict formula), the patient will be assigned to one of the different diet energy levels, depending on their needs (From 1600 to 3100 Kcal daily): 1600, 1800, 2000, 2200, 2500, 2800 and 3100 Kcal.

The patients will talk about the diet record they completed during the selection phase with the dietician.

Each patient will be given a diet record for 3 days, two working days and one holiday, which they will complete and return at the next visit (S4) (Annex 6). Furthermore, they will be reminded of the instructions on how to complete the record as precisely as possible, as indicated in the methodology section.

- Medication

The medication necessary for the period from S0 until the next visit in S4 will be issued according to the randomisation number assigned. The patient will be informed as to when to take it and how to return it.

Visit S4 (Day 28± 3)

The participants will go to the Hospital and the visit will comprise several aspects:

- *Clinical*

Identify the presence of clinical problems, concomitant treatments, etc.
Collection of adverse events

Blood pressure and weight will be taken.

- *Analytical*

A request for two analyses will be issued. For the blood extractions, the patient will go to the hospital during the week prior to visit S8 on two occasions, with a 3- or 4-day interval between them, and the last extraction will be performed at most 3 days before the following visit. In this way, the investigator will have both results, particularly for the lipid profile, at the S8 day visit.
Only the lipid profile will be determined at the first extraction. At the second extraction, and besides the lipid profile, the same biochemistry and haematological parameters as at visit S0 will be requested. Genetic polymorphisms will not be determined.

- *Dietary*

The patient will return the diet record delivered at week S0.

A new 3-day diet record to be returned at the following visit will be provided. (S8).

- *Medication*

Evaluate compliance with treatment with fibre. The patient should return used and unused medication containers.

The investigator will deliver the medication for the following treatment period until the next visit in week 8.

Visit S8 (Day 56± 3)

The participants will go to the hospital for a visit addressing the following aspects:

- *Clinical*

A clinical history similar to that of S0 will be made

Identify the presence of clinical problems, concomitant treatments, etc.

Collection of adverse events.

Blood pressure and weight will be taken.

- *Analytical*

The results of the mean LDL-C value obtained in the two analyses made in the previous week (S7), at most 3 days before visit S8, will be noted. Similarly, the values obtained in the analyses on the last day of S7 will be noted as final parameters of the biochemistry and haematological trial.

- *Dietary*

The patients will already have returned the 3-day record the day they went to the hospital for the second blood sample extraction.
The dietician will address the diet follow-up and possible deviations. If the patient has to continue in the study he/she will be given diet guidelines according to his energy needs.

- *Medication*

Evaluate compliance with treatment with fibre. The patient should return used and unused medication containers.

Two situations may arise at this point: 1) The patient has reached the therapeutic objective (LDL-C < 130) and will end the study, or 2) the patient will present an LDL-C ≥ 130 mg/dl and will continue with the same treatment with a statin (Simvastatin) added for a further 8 weeks.
The instructions on how to administer the fibre, the Simvastatin and, as the case may be, the patient's regular medication will be gone over once again.

If the patient is to continue in the trial, the investigator will issue the same medication that the patient was taking + a statin in sufficient quantity for 4 weeks until visit S12.

Fibre + statins phase

Visit S12 (Day 84 ± 3)

The participants will go to the hospital for a visit addressing the following aspects:

- *Clinical*

Identify the presence of clinical problems, concomitant treatments, etc.

Collection of adverse events.

Blood pressure and weight will be taken.

- *Analytical*

A request for two analyses will be issued. For the blood extractions, the patient will go to the hospital during the week prior to visit S16 on two occasions, with a 3- or 4-day interval between them, and the last extraction will be performed at most 3 days before the following visit. In this way, the investigator will have both results, particularly for the lipid profile, at the S16 visit.
Only the lipid profile will be determined at the first extraction. At the second extraction, and besides the lipid profile, the same biochemistry and haematological parameters as at visit S0 will be requested. Genetic polymorphisms will not be determined.

- *Dietary*

The patient will return the 3-day record and will be given a new one to complete.

- *Medication*

Evaluate compliance with treatment with fibre + statin. The patient should return used and unused medication containers.

The investigator will issue medication for the 4 remaining weeks of the study.

Visit S16 (Day 112 ± 3)

The participants will go to the Hospital for the final visit of this second period, addressing the following aspects:

- *Clinical*

A clinical history similar to that of S0 will be made.

Identify the presence of clinical problems, concomitant treatments, etc.

Collection of adverse events.

Blood pressure and weight will be taken.

- *Analytical*

The results of the mean LDL-C value obtained in the two analyses made in the previous week (S15) will be noted at this visit S16. Similarly, the values obtained in the analyses on the last day of S15 will be noted as final parameters of the biochemistry and haematological trial.

- *Dietary*

The patient will return the 3-day record.

- *Medication*

Evaluate compliance with treatment with fibre + statin. The patient should return used and unused medication containers.

8.3. Methodology

8.3.1. Laboratory analysis

The biochemistry analyses and haemogram will be carried out at each centre, barring the lipid profile, which will be conducted twofold, i.e. it will be determined in each centre so as to have the results in a few days and enable the investigator to take the relevant decisions at each point in the trial (inclusion criteria, continue or not at S8). Moreover, the determination of all samples will be centralised so as to minimise variability between centres and between analytical techniques.

The analysis of genetic polymorphisms will be centralised.

The trial central laboratory will be in the Hospital Universitario San Joan de Reus.

For these centralised determinations to be conducted, the samples should be stored frozen (–20ºC) and sent to the central laboratory in accordance with the procedure explained in Annex 8.

- ***Cholesterol and triglycerides:***
  They will be determined by means of an enzymatic colorimetric reaction adapted to a centrifugal autoanalyser.

- ***. HDL cholesterol:***

A polyethylene glycol solution will be used, precipitating the lipoproteins containing the apolipoprotein B (LDL and VLDL), making it possible to recover total HDL in the supernatant.

- ***. LDL Cholesterol:***
  It will be calculated by means of the Friedewald's formula:
  c- LDL(mg/dl) = total cholesterol – HDL-C – (triglycerides/5)

- ***. Vitamins A, E and C***
  They will be determined by high performance liquid chromatography (HPLC) following a specific treatment depending on whether they are liposoluble (A and E) or hydrosoluble (C).

- ***. Minerals***
  They will be determined by colorimetry (Fe, Ca) or atomic absorption spectrophotometry (mg).

- ***. Biochemistry and haemogram***
  They will be determined by means of an autoanalyser.

- ***In vivo* study of the cholesterol-lowering, antioxidant, anti-inflammatory and antithrombotic activity**

Obtaining of the sample*:*
All patients will have 20 ml of blood extracted into EDTA plasma tubes (1 mg/ml). Immediately after extraction, it will be centrifuged at 1000 g at 4ºC. The plasma will be separated and 10 μM of BHT will be added in order to protect the sample from lipid peroxidation. The plasma will be aliquoted and stored at –20ºC for later analysis of the oxidative parameters (period of no more than 6 months). A fresh aliquot of plasma will be used for the determination of the biochemistry profile.

In Oxidation and antioxidant markers *:*
Determination of vitamin E: The vitamin E content (α-tocopherol, γ-tocopherol) in plasma and LDL will be determined using high resolution liquid chromatography (HPLC; Hewlet Packard 1050). Briefly, the internal standard (50 mg/l of tocopherol acetate) in ethanol will be added to 100 μl of sample (plasma EDTA, 10μM BHT) and after the precipitation of the proteins the lipids will be extracted with n-hexane. Immediately before injecting the sample into the HPLC, the lipid residue will be reconstituted in 100 μl of ethanol. The mobile phase is methanol (100%); the column used is Spherisorb-ODS 2, 125 × 4mm, ∅5 μ and the detection of α-tocopherol and g-tocopherol is carried out at 292 nm. The concentration is determined extrapolating the results of the sample to a standard curve of known concentrations (1-25 mg/l) and correcting the loss of vitamin due to the process of extraction by the internal standard. The plasma vitamin E concentrations are expressed in mg/l.
The kinetics of oxidation (conjugated dienes) of the plasma lipoproteins LDL and HDL will be determined monitoring the change in absorbance at 234 nm at 30ºC with a UV spectrophotometer (Kontron) in the presence of 6.7μM Cu^{++}.

The determination of oxidated LDL in plasma will be done by an ELISA procedure using the murine monoclonal antibody mAB-4E6 as capturing antibody and an antibody against the oxidated Apo B joined to the peroxidase in solid phase. The coefficients of variation intra- and inter-trial for the methodology are: 2.8 % and 7.3 %, respectively.
The lipid peroxides in plasma will be measured using the generation of adducts of malondialdehyde-thiobarbiturate at 532 nm, expressed as equivalents of malondialdehyde, after lipid extraction with chloroform-methanol. The coefficients of variation intra- and inter-trial for the methodology are: 4.2% and 6.8 %, respectively.
In plasma, the concentrations of total cholesterol, LDL, HDL and triglycerides, apoliproteins A and B, and lipoprotein (a) will be determined using the standard test and autoanalyser.

Inflammation markers*:*
The plasma concentrations of IL-1beta, interleukin-6, TNF-alpha, amyloid-A, C-reactive protein, fibrinogen will be determined using commercial ELISA kits (Amersham). The leukocyte concentration will be determined using automatic autoanalyser.

Indicators of antithrombotic activity:

The plasma concentrations of folic acid, homocysteine, plasminogen activator inhibitor (PAI-1) will be determined using commercial ELISA kits (Amersham).

- **Determination of the polymorphisms of the genes which predispose to cardiovascular diseases.**

Extraction of DNA:

The obtaining of genome DNA from each subject will be carried out using the cell package according to the protocol described in the commercial kit (Servicios Hospitalarios [Hospital Services], Spain).

Detection of the polymorphisms of the Apolipoprotein E (ApoE), angiotensin-converting enzyme (ACE), plasminogen activator inhibitor-1 (PAI-1) and fibrinogen genes*:*

A region which contains the described polymorphisms of the Apo E (E3/E3, E3/E4 and E4/E4), ACE (insertion/deletion (I/D)), PAI-1 (4G/5G promoter) and fibrinogen(-455G/A) genes will be amplified by PCR. The presence of the genetic variation will be evaluated using enzyme restriction.

8.3.2. Diet analysis

The participants will be advised to follow a diet according to the recommendations for the prevention and treatment of cardiovascular diseases. Polyunsaturated fatty acids will account for less than 7% and the rest will be provided by monounsaturated fatty acids. Cholesterol intake will be lower than 200 mg daily.

The percentage of saturated fatty acids during the selection period (S-2 to S0), will be a maximum of 12%. Once the patient is randomised (after S0), the percentage of saturated fatty acids should be ≤ 7%.

Simultaneously, the intake of carbohydrates will be 55%, and proteins will provide approximately 15% of the remaining calories.

Alcohol intake will be lower than 30 g daily (equivalent to 1-3 glasses of wine or 1-3 beers or 1 standard measure of distilled drinks) and half for women.

The diets will be isocaloric and will be assigned according to needs, to maintain a stable participant weight.

To facilitate compliance with diet recommendations in participants from the different centres, the diet will be predefined in 7 levels of calories from 1600 Kcal to 3100 Kcal daily, to be able to adapt them to the energy needs of each participant. Similarly, cyclic 7-day menus will be made up, with a total of 14 meals, specifying the quantity of the different foods, as well as the diet characteristics. Each participant will be given their specific diet at an individual visit with the dietician.

In order to achieve maximum precision in the calculation of food intake, the volunteers will receive training on how to make measurements at home during the first interview with the dietician. The way the usual measures are assessed will be checked at the following visits to the dietician.

Compliance with the diet will be monitored by means of 3-day diet records and an interview with the dietician at the time of a medical visit. Furthermore, in the other weeks of the intervention period, the participants will be interviewed by telephone, without notice, for a 24h diet reminder (Annex 7).
Body weight will be monitored in parallel at each visit. If the participants' weight differs by more than 1 kg the calorie intake will be modified.

The participants will be encouraged to continue with their regular physical exercise and lifestyle during the study, and any change must be notified to the investigators.

The conversion of food into nutrients will be made using the French food composition tables (Répertoire géneral des aliments).

The procedure for the calculation, in the study Case Report Form, for the record of dietary and energy requirements, will be done as specified in Annex (16).

8.3.4. Treatment compliance

The investigator will monitor treatment compliance by counting the medication sachets/tablets returned by the patient at each visit.

8.3.5. Collection of adverse events

Possible adverse events will be recorded at all controls. See section 9 of the protocol.

9. ADVERSE EVENTS

9.1. General Information

An Adverse Event (AE) is any untoward medical event that befalls a clinical research patient or subject treated with a medicinal product, which does not necessarily have a causal relationship with the use of the treatment. An AE is therefore any unfavourable and unintentional sign (including an abnormal laboratory finding), symptom or disease temporarily associated with the use of a medicinal product (research or marketed), whether or not it is regarded as related to the product. An Adverse Event (AE) is any clinically significant disease, sign, symptom or abnormal laboratory finding which has appeared or deteriorated during the course of a clinical trial, regardless of its causal relationship with the investigational drug. The collection of information on non-serious AE should begin following the first administration of an investigational product. Serious AEs should begin to be collected once the subject's informed consent to participate in the study has been obtained.

AEs may be reported spontaneously or during the interview and physical examination of a subject. All AEs identified should be noted and described in the relevant page of Serious or Non-serious AEs in the CRF. If the diagnosis of the disease or underlying disorder is known, it will be included instead of the individual symptoms.

Subjects who have AEs that lead to the interruption or suspension of treatment with the investigational product, or those who have AEs that are maintained at the end of their participation in the study, should be monitored, as applicable. If possible, the result of any AE leading to permanent suspension or which is present at the end of the study will be reported, particularly if the AE was regarded by the investigator as definitely, probably or possibly related to the investigational product.

9.2 Non-serious Adverse Events (NSAEs)

Any AE not classified as serious, as defined below, must be noted on the non-serious AE page of the CRF. These AEs will be followed up until they are solved or stabilise, and will be reported as SAEs if they become serious.

9.3 Serious Adverse Events (SAEs)

A serious adverse event should be logged on the Serious Adverse Events (SAE) page of the CRF and should be communicated promptly to comply with the requirements of the regulatory authorities.

A Serious Adverse Event or Serious Adverse event is any untoward medical event which, at any dose:

- Causes death
- Is life-threatening (defined as an event in which the life of the patient or subject were endangered at the time of the event, and does not refer to an event that could have been fatal had it been more serious)

- Requires or prolongs hospitalisation
- Leads to a persistent or significant disability/incapacity
- Is cancer
- Is a congenital anomaly or birth defect
- Is an overdose (defined as accidental or intentional intake of any dose of a product that is regarded as excessive and medically important),
- Leads to dependence or drug abuse
- Is a major medical event (defined as a medical event which, while perhaps not immediately life-threatening, may be fatal or lead to hospitalisation, but which based on a suitable medical or scientific judgement may compromise the patient or subject or may require intervention [e.g. medical, surgical] to prevent a serious consequence, as defined above. Examples of major medical events will include, although this list is not to be regarded as exhaustive, intensive treatment of allergic bronchospasm, either in the emergency department or at home, as well as blood dyscrasia or convulsions that do not require hospitalisation.

To comply with the requirements of the regulatory authorities, adverse events classified as "serious" must be handled quickly and reported immediately to the Person in charge of Pharmacovigilance at Madaus.

Any serious AE which in the opinion of the investigator, is definitely, probably, possibly, probably not related or not related to the investigational product must be notified by telephone, wherever possible. Nevertheless, all serious AEs, whether or not they are related to the investigational product, should be reported immediately by fax, and the completed SAE report sheet subsequently sent by mail, as recorded in the CRF. Transmission by fax does not replace the obligation to send the SAE report sheet by mail. A courier service may be used instead of fax. A follow-up report will be required if only limited information is initially available.

CONTACTS FOR THE NOTIFICATION OF SAEs IN SPAIN

For the notification of SAEs, the investigator should contact the assigned monitor at the centre in order to inform him or her of the event. The monitor will advise the investigator of the procedure to follow for the sending of information. If it is not possible to contact the monitor, the investigator may contact the Madaus Pharmacovigilance service directly (see contact below).

Dr. Anna Anguera Vilá
Servicio de Farmacovigilancia
Tel: 93 2988206
Fax: 93 4319885
e-mail: aanguera@madaus.es

Madaus, as the sponsor of the trial, will notify the Spanish Medicines Agency (AEM), competent organs of the Autonomous Community and the Independent Ethics Committees (IEC) where the trial is being conducted of any important information affecting the safety of the investigational medicinal product. This communication will

be made according to the criteria specified below. (Circular 15/2001 of the AEM on clinical trials with medicinal products):

9.4. Notification of Adverse Events to the Health Authorities

Prompt notification to the Spanish Medicines Agency

The sponsor will notify the AEM *of all serious and unexpected events* that may be related to the investigational medicinal product (suspicion of serious and unexpected adverse event) whether they have occurred in Spain or in other countries, and whether they have occurred in the authorised clinical trial or in other clinical trials or in a different context of use. However, for medicinal products marketed in Spain used in the authorised conditions, only the suspicion of serious and unexpected adverse events that occur in the actual trial, either inside or outside Spain, if the trial is multinational, will be reported.

The maximum term of notification will be 15 calendar days as of the moment they become known. When the suspicion of serious and unexpected adverse event has caused the death of the subject, or threatened his life, the sponsor will notify the AEM within a maximum term of 7 natural days as of the moment the sponsor learns of the case. This information should be completed, as far as possible, within the following 8 days. For adverse events that occur in clinical trials performed in Spain, the number assigned by the AEM to the corresponding protocol should be mentioned, as well as the PEI (Spain's equivalent to Investigational New Drug, IND) number, as applicable. In the case of adverse events that occur outside Spain, reference will be made to the number assigned by the AEM to the PEI and/or clinical trials affected.
When this type of suspicion of adverse events occurs in a double-blind trial, the specific patient treatment code should be unmasked for the purpose of notification. Whenever possible, blinding shall be maintained for the investigator and for the person in charge of the analysis and interpretation of the results, as well as drawing up the study conclusions. In cases where this system of notification may be considered to interfere with the validity of the study (for example, when the primary endpoint is the occurrence of death) a specific system of notification may be agreed to with the AEM.

The suspicion of adverse events ascribable to the placebo will not be subject to this individualised system of notification.

Notifications should be addressed to the *Subdirección General del Medicamentos de Uso Humano* (Subdirectorate General of Medicinal Products for Human Use) by fax if the adverse event occurs in Spain: (+34 91 596 40 69) or else by post or delivered personally to the *Unidad de Registro y Tasas* (Registration and Fees Unit) of the AEM: (Paseo del Prado 18-20 – 28014 Madrid) if they occur outside Spain. Only Spanish, the official language of the State, will be accepted for notifications of adverse events that occur in Spain. English will also be accepted for those occurring outside Spain. The notification form given in Annex I of this circular will be used for

notifications of suspicion of adverse events that occur in Spain. The CIOMS I form may be used for notifications of suspicion of adverse events that occur outside Spain.

In the case of *marketed medicinal products used according to the authorised conditions* (including the control medicinal product), these notifications will be sent to the Division of Pharmacoepidemiology and Pharmacovigilance of the AEM (not to the Subdirectorate General of Medicinal Products for Human Use). If the adverse events occur in Spain they should be faxed (+34-91-596 78 91). They will only be accepted if they are written in Spanish, the official language of the State. Any adverse events occurring outside Spain should only be sent by Post or delivered personally to the Registration and Fees Unit of the AEM (Paseo del Prado, 18-20 - 28071 Madrid). They will also be accepted in English. The notification form given in Annex I of this circular will be used for notifications of suspicion of adverse events that occur in Spain. The CIOMS I form may be used for notifications of suspicion of adverse events that occur outside Spain.

Prompt notification to the IEC and to the competent organs of the Autonomous Communities

The sponsor will notify the IEC and competent organs of the Autonomous Communities where the trial is being conducted (see list in ANNEX A), individually and within the maximum term of 15 days, of all the suspected adverse events which are serious and unexpected and related to the investigational product occurring in patients recruited in their respective areas (or in other words, notify the IEC of reactions corresponding to the patients recruited in their health centre or area and notify the Autonomous Communities of reactions occurring in patients recruited in the Autonomous Community in question). This deadline will be 7 days for suspected adverse events that lead to death or are life-threatening.

The sponsor will report any other information on serious and unexpected adverse events associated with the investigational medicinal product (that occur in Spain or outside Spain) if so requested by the IEC when the protocol is reported, if so required by the specific regulations of the Autonomous Communities and in any case if the information represents a substantial change in the safety profile of the investigational product.

The reporting of safety information by the sponsor to the investigators will comply with the specifications in the ICH Good Clinical Practice guidelines.

Safety reports

In addition to prompt notification, clinical trial sponsors will prepare a report evaluating the safety of the investigational medicinal product, taking all the available information into account. This report will be submitted to the AEM (Subdirectorate General of Medicinal Products for Human Use), to the competent organs of the corresponding Autonomous Communities and to the IEC that gave a favourable report to the trial, annually until the end of the trial and whenever requested by the Health Authorities or the IEC. It will include a list with all the suspected serious

adverse events reported inside and outside Spain, classified by organs and systems following the MedDRA classification, and an evaluation report will be made on the safety of the product for the trial subject (in the case of marketed products used in the authorised conditions, this list will only refer to suspected serious adverse events occurring in the clinical trial in question). It will also specify, as the case may be, the measures proposed to minimise the risks found. Finally, explicit justification will be provided of the possible need to modify or renew the informed consent, as well as the Investigator's Brochure. This report will not replace the application of amendments to the protocol, which will continue with its specific procedure.

Without prejudice to the frequency of safety reports indicated, the sponsor will prepare an *ad hoc* evaluation report whenever there is a relevant safety problem. This report will be submitted without delay to the AEM, the competent organs of the Autonomous Communities and the IEC concerned.

The safety report may be a part of the corresponding annual and final reports or be prepared separately. In any case, the annual and final reports will contain all the adverse events, in tabulated form, detected in the trial up until the time of the preparation of the report.

The safety reports, as well as the annual and final reports, should be written in Spanish, the official language of the state.

9.5. Abnormal laboratory findings

Besides being recorded on the relevant laboratory test results pages of the CRF or being sent electronically to a central laboratory, any result of a laboratory test that fulfils the serious adverse event criteria (see Section 9.3) should also be recorded on the SAE page of the CRF so that the Person in charge of Pharmacovigilance can collect additional information on the abnormal finding, including information on its relationship with the investigational product or other causes, action taken and resolution.

9.6. Other Safety Considerations

Any clinically significant change recorded during the intermediate or final physical examinations, electrocardiograms, radiographies or other potential safety evaluations, whether these procedures are required by the protocol or not, must also be recorded on the appropriate AE page (that is, NOT SERIOUS or SERIOUS) of the CRF so that the Person in charge of Pharmacovigilance can collect additional information on the abnormal finding, including information on its relationship with the investigational product or other causes, action taken and resolution.

9.7. Imputability criteria

The following imputability criteria are used in this study:

- **Certain** when there is a reasonable causal relationship between the investigational drug and the SAE. The episode improves with the withdrawal of the investigational drug and reappears when the drug is resumed, provided it is clinically viable.
- **Probable** when there is a reasonable causal relationship between the investigational drug and the SAE. The event improves with the withdrawal of the investigational drug. Treatment need not be restarted.
- **Possible** when there is a reasonable causal relationship between the investigational drug and the SAE. There is insufficient information on the withdrawal of the investigational drug or it is not very clear.
- **Improbable** when there is a temporary relationship with the administration of the investigational drug but there is no reasonable causal relationship between the investigational drug and the SAE.
- **Not related** when there is no temporary relationship with the administration of the investigational drug (too early, late or not taken) or there is a reasonable causal relationship between the SAE and another drug, intercurrent disease or circumstance.

The categories of *certain, probable* and *possible* are regarded as being related to the investigational drug. The categories of *improbable* and *not related* are regarded as not being related to the investigational drug.

The expression “reasonable causal relationship” means that there are facts (evidence) or arguments that suggest a causal relationship. If the information on the SAE is insufficient or contradictory, the event should be regarded as possibly related until further information is available. Since these events may be promptly reported, each investigator should do his utmost to collect all the extra information necessary.

10. ETHICAL CONSIDERATIONS

10.1. General considerations

This trial will be conducted according to the of Declaration of Helsinki, the Good Clinical Practice guides of the ICH (International Conference of Harmonization), Madaus, S.A.'s SOPs, and Quintiles' and the legislation in force (Royal Decree on clinical trials 223/2004).

10.2. Data Confidentiality

The investigator will guarantee that all the persons involved observe the confidentiality of any information on the trial subjects (Article 37 R.D. 223/2004).

All the parties involved in a clinical trial will observe the strictest confidentiality to ensure that the personal or family privacy of the subjects participating in it is preserved. Similarly, suitable measures should be taken to avoid the access of unauthorised personnel to the trial data (Article 3 R.D. 223/2004).

The treatment of the personal data of the subjects participating in the trial, particularly with regard to consent, will comply with the provisions of the Constitutional Law 5/1992 of 29 October (Article 3 R.D. 223/2004) and European Directive on Data Protection (95/46/EC).

The data obtained in this study will be checked by a monitor appointed by the sponsor and will be used exclusively to draw scientific conclusions. The identity of the patients is confidential and will be known only by the investigator and his collaborators, the auditors and monitors appointed by the sponsor and the inspectors of the competent authorities.

10.3. Trial Insurance

The sponsor, Madaus, S.A., has taken out a public liability insurance policy for a maximum sum of 250.000 € per subject included in the clinical trial (art. 8 RD 223/2004) (Annex 10)

The insurance will cover all the liabilities of the sponsor, the investigators and his collaborators and the proprietor of the Hospital or Centre where the trial is conducted (Art. 8 of the RD 223/2004).

It is assumed, unless proven otherwise, that injuries affecting the health of the person involved in the trial, during the trial and in the period of one year following the end of the treatment, took place as a result of the trial. However, after one year has elapsed the trial subject is required to prove the link between the trial and injury or damage.

10.4. Independent Ethics Committee (IEC)

Before the study begins, the sponsor and the investigator should obtain the written approval or favourable opinion of the IEC for this protocol, the Informed Consent Form, the material used for patient recruitment (e.g. announcements) as well as any written information provided to the patients. Furthermore, they should provide the IEC with the Investigator's Brochure or Summary of Product Characteristics, the information sheet given to the subject and any update.

Madaus, as sponsor, should send reports, updates and any other information to the IEC (e.g. Safety Reports, Amendments to the Protocol, Administrative Letters) in accordance with the requirements of the regulatory authorities or procedures of the institution (R.D. 223/2004).

10.5. Informed consent

The preparation of the Informed Consent Form is the Investigator's responsibility. It must include all the elements required by the ICH, GCP and the applicable regulatory directives, and should comply with Good Clinical Practices and the ethical principles promoted by the Declaration of Helsinki (Annex 9). The consent form should also include a declaration that the sponsor and the health authorities may have direct access to the subjects' records. Before the study begins, the written approval/favourable opinion of the IEC on the Informed Consent Form and any other information provided to the subjects should be obtained.

The subject will preferably give his consent in writing, or else orally before witnesses independent of the investigating team, who will be present during the discussion of the informed consent and will declare on their own responsibility. After the subject has given his oral consent, the witnesses should personally sign and date the Informed Consent Form indicating that the information provided is accurate, and that the subject has understood it and given his consent freely (Annex 4).

The Investigator should provide the subject or his legally authorised representative with a copy of the consent form and of the Patient Information Sheet in a non-technical and easy-to-understand language. The Investigator should give the subject or his legally authorised representative time to ask questions on the details of the study. Then, the subject or his legally authorised representative and the person who participated in the discussion of the informed consent will sign and date the informed consent personally. The subject or his legally authorised representative should receive a copy of the Informed Consent Form and any other written information provided to the subject on the study before participating in the study.

The informed consent and any other information provided to the subject or to his legally authorised representatives should be revised whenever new information becomes available and which is relevant to the voluntary participation of the subject, and should be approved or given the favourable opinion of the IEC before it can be implemented. The Investigator, or the person appointed by him, should inform the subject or his legally authorised representative comprehensively on all the relevant

aspects of the study, and on any new information that may affect the subject's disposition to continue to participate in the study. This communication must be documented.

During the participation of a subject in the trial, any update of the consent form or of the Patient Information Sheet should be provided to the subject.

The subject participating in a clinical trial or his representative may withdraw his consent at any time, without having to account for their decision and without this entailing any liability or prejudice to them (art. 7.5 RD 223/2004).

10.6. Confidentiality

The investigator will guarantee that all the persons involved observe the confidentiality of any information on the trial subjects (Article 3.2 R.D. 223/2004).

All the parties involved in a clinical trial will observe the strictest confidentiality to ensure that the personal or family privacy of the subjects participating in it is preserved. Similarly, suitable measures should be taken to avoid the access of unauthorised personnel to the trial data (art. 3 R.D. 223/2004).

The treatment of the personal data of the subjects participating in the trial, particularly with regard to consent, will comply with the provisions of the Constitutional Law 15/1999 of 13 October (Article 3.2 R.D. 223/2004) and the European Directive on Data Protection (95/46/EC).

The data obtained in this study will be checked by a monitor appointed by the sponsor and will be used exclusively to draw scientific conclusions. The identity of the patients is confidential and will be known only by the investigator and his collaborators, the auditors and monitors appointed by the sponsor and inspectors of the competent authorities.

10.7. Trial Budget

See Annex 15.

10.8. Insurance policy

The Spanish Legislation requires coverage for subjects participating in clinical trials with a public liability policy (Article 8, R.D. 223/2004). The study sponsor, Madaus, has taken out this policy, in accordance with the legislation in force (Annex 10).

11. PRACTICAL CONSIDERATIONS

11.1. Responsibilities of all the participants

11.1.1. Compliance with the protocol and amendments to the protocol

The study will be carried out as described in this approved protocol. All revisions should be discussed and revised by the investigator and the Coordinator.

Any amendment to an already authorised clinical trial protocol must be reported to the Independent Ethics Committees (IEC) involved in it, to the Spanish Medicines Agency and the Autonomous Communities. When the amendment is relevant, a prior report by the IEC involved in the trial and authorisation by the Spanish Medicines Agency will be required. Relevant amendments will be those that entail an increased risk for the subjects participating (Article 25 R.D. 223/2004).

The investigator may not implement any deviation or change in the protocol without the prior revision and documented approval/favourable opinion by the IEC regarding any such amendment, except for any changes whose purpose is to remove an immediate risk for the study subjects. Any significant deviation from the protocol should be documented in the case report form.
If a deviation or change in the protocol is implemented before the approval/favourable opinion of the IEC is secured, in order to remove an immediate risk, any such deviation/change shall be submitted, as soon as possible, to:

- the IEC for its revision and/or favourable opinion;
- the Spanish Medicines Agency.

The documentation pertaining to the approval signed by the president or the person designated by the latter in the IEC should be sent to the sponsor.

If the revision is an administrative letter, it should be submitted to the IEC, to the Spanish Medicines Agency and to the Autonomous Communities for their information.

If an amendment substantially alters the study design or increases the potential risk for the subject: 1) The Informed Consent form should be revised and be resubmitted to the IEC for revision and approval/favourable opinion; 2) The revised form should be used to obtain the consent of the subjects already included in the study if they are affected by the amendment; 3) the new form should be used to obtain the consent of new subjects before recruitment.

The revised form should also be sent to the Spanish Medicines Agency (Article 25, R.D. 223/2004).

11.2. Control of compliance in the protocol

The sponsor's representatives should be allowed to visit all the study centres periodically to check data integrity and to validate the quality and the veracity of the study. The study documents will be revised at the centre and compared with the original documents, talks will be held with the investigator on the progress of the study and the suitability of the facilities will be assessed constantly.

Furthermore, the study may be evaluated by the sponsor's internal auditors and by the inspectors designated by the Health Authorities, who will be given access to the CRFs, original documents and other study archives. The reports of the audit carried out by the sponsor will always be kept confidential.

The investigator should notify the sponsor quickly of any scheduled audit by any health authority, and promptly send copies of the audit report to the sponsor.

11.3. Data filing

11.3.1. Files and reports

The Investigator should prepare and maintain suitable and accurate records designed to log all the observations and other data relevant to the research in each subject treated with the investigational product or included as a control in the trial.
The investigator will be responsible for ensuring that the data are duly recorded in the electronic Case Report Form for each patient and that they are consistent with the original documents, or in the case of discrepancy an explanation should be provided.

The subjects should be identified by their initials, date of birth and by the patient's number, as applicable. All the information required should be included in the CRF in the spaces provided for this purpose. If a datum is not available or is not applicable, it should be documented as such; no blank spaces should be left.

Each investigator will sign the electronic case report forms in order to ensure that the observations and findings have been recorded fully and accurately. The investigator will maintain a sheet of signatures to document the signatures and initials of all the people authorised to input or correct data in the CRFs.

The confidentiality of any documents that might identify the subjects must be protected, observing their privacy and the rules of confidentiality in accordance with the applicable regulatory requirements.

This protocol will use the software belonging to “Quintiles” and any updates which may be made known during the course of the trial, in order to record data electronically at the study site and to be able to revise the data electronically.

11.3.2. File storage

The sponsor is responsible for the storage of the trial dossier. The investigator will keep the patient identification codes for at least 15 years after the conclusion or interruption of the trial. The clinical records of the patients and other original data will be stored for the maximum period of time allowed by the Hospital, institution or centre where the trial is carried out

The investigator should contact the sponsor before destroying any record associated with the study. The sponsor will notify the Investigator as soon as it is no longer necessary to keep the trial archives.

Should the investigator withdraw from the study (for example, transfer, retirement), the records should be transferred to another person appointed by mutual agreement (for example, another investigator, IEC). Written notification of this transfer should be sent to the sponsor.

11.4. Samples for clinical research and labels

The medication will be provided by the trial sponsor, duly labelled according to the applicable standards.

The pharmacy service, and in exceptional cases the investigator, are responsible for ensuring that an updated Register of the availability of the investigational drug is kept at each Investigation Centre where the investigational product is registered and stored.
The records or notebooks should comply with the applicable regulations and directives and should include:

- Amount received and located in the storage site
- Amount currently stored
- Label ID number or batch number
- Dates and initials of the person responsible for entering and removing drugs from the inventory
- Amount dispensed and returned by each subject, including the subject's unique identifier
- Amount transferred to another area for dispensing or storage
- Use of medication outside the study (e.g. due to losses, wastage, damage)
- Amount returned to the sponsor
- Amount destroyed at the investigation centre, as applicable
- Samples sent to third parties for bioavailability/bioequivalence, as applicable

The sponsor will provide forms to facilitate inventory control if the investigation centre personnel do not have a system fulfilling these requirements in place.

Once the study has been completed or terminated, all unused and/or partially used drugs should be returned to the sponsor if the centre is not authorised to destroy them.

11.5. Publication policy

In order to prevent the premature communication of information and patentable inventions, the publication of the results will be performed by common agreement with all the investigators and the sponsor.

All publications, abstracts, communications at congresses, etc., will be signed by the principal investigator. In the case of multicentre studies, the order of the signatories will depend on the number of patients included at each centre, barring the principal investigator, who will sign first, or the person designated by him to draft the text to be published.

12. STATISTICAL ANALYSIS

12.1. Descriptive analysis

The description of the endpoints studied will be performed according to conventional techniques.
The quantitative endpoints will be described using means, standard deviations, quantiles and outliers, whereas the qualitative endpoints will be described by means of frequency tables.

The normality of the quantitative endpoints will be evaluated by the usual normality tests (Kolmogorov-Smirnoff)
If a normal distribution and homoscedasticity are assumable in the values of an endpoint, parametric analysis procedures will be used in subsequent inferential studies.
Non-normal distribution endpoints will be transformed as necessary to normalise the distribution of their values. If no transformation that meets the premises of normality is found, equivalent non-parametric procedures will be used alternatively in the analysis of this endpoint.

12.2 Homogeneity of samples

For visit S0, a determination will be made to establish whether the patients of both treatment groups are homogeneously distributed in terms of age, gender, lipid profile, mineral plasma levels, vitamin plasma levels, blood test parameters, weight and genetic polymorphisms.
For the quantitative variables, the homogeneity of the samples will be evaluated by the Student's *t* test if the normality premises are met; otherwise the Mann-Whitney non-parametric test will be used. The Chi square test will be used for qualitative endpoints.
In all cases, the null hypothesis considered will be the non-existence of differences between the two treatments for a two-tailed level of significance of 0.050.

12.3 Efficacy analysis

12.3.1 Primary efficacy analysis.

The primary efficacy analysis will be conducted to evaluate the effect of the treatment with *Plantago ovata* husk versus placebo with regard to plasma LDL-C concentrations in patients with moderate hypercholesterolemia with low saturated fat diet.

The changes in the values of the plasma LDL-C concentrations will be evaluated by creating two endpoints:

- The primary analysis of the primary evaluation will be carried out with the total difference in the concentrations of LDL-C in plasma between visit S8 and visit S0 between both study groups.

- As additional sensitivity measure, the percentage of reduction of the concentrations of LDL-C in plasma between visit S8 and visit S0 between both study groups will also be evaluated.

The principal working hypothesis will be:

H_0: Δ LDL *plantago ovata* - Δ LDL placebo < $|7.5|$ mg/dl

H_1: Δ LDL *plantago ovata* - Δ LDL placebo $\geq$ $|7.5|$ mg/dl

Where Δ LDL = final LDL – baseline LDL

The statistical analysis will be carried out using a covariance analysis (ANCOVA) model with the baseline value as co-variable:

$$Y_{it} = \mu + Y_{itBASELINE} + T_t + e_{it}$$

where,

Y_{it}	indicates the value of variable Y (difference in concentrations between visit S8 and visit S0) measured for patient I receiving treatment T.
$Y_{it\ BASELINE}$	indicates the baseline value of the variable Y (difference in concentrations between visit S8 and visit S0) measured for patient I.
μ	indicates the overall average
T_t	indicates the fixed effect of the treatment (T = 1 a 2)
e_{it}	indicates residual error, where $e_{it} \sim N(0, \sigma_e^2)$

In order to study other variables at other post-baseline visits, when necessary, an ANCOVA model will be made for each one, analogous to that described above. The baseline homogeneity will be evaluated using a Student's *t* test for independent data.

The variation in the LDL-C concentrations for week 8 to week 16, will be measured by way of intragroup analysis using analysis methods for repeat measurements.

The level of statistical significance will be established at 0.05 bilateral.

The evaluation of the continuous secondary efficacy endpoints will be analogous with the previously described ANCOVA for the primary endpoint. The continuous secondary endpoints to be analysed are described below:

- Weight and blood pressure
- Total cholesterol, triglycerides, HDL cholesterol, apolipoproteins (apo) B-100 and A-1 and lipoprotein (a).
- The oxidation markers: alpha tocopherol and gamma tocopherol in LDL, resistance of LDL to oxidation (lag time), LDL oxidation "in vivo" and lipid lipoperoxides.
- The inflammation markers: IL-1beta, interleukin-6, TNF-alpha, amyloid-A, high sensitivity C-reactive protein, fibrinogen, leukocytes.

- The indicators of antithrombotic activity: folic acid, homocysteine, plasminogen activator inhibitor (PAI-1).
- The individual response to the plasma lipids and their modulation by apoliprotein E polymorphisms, cholesterol ester transport protein (CETP), apolipoprotein A-V, FABP 2, ABCG5 and ABCG8, fibrinogen, fibrinogen activator inhibitor, angiotensin converting enzyme and other polymorphisms which might describe their involvement in the development of cardiovascular diseases.

- Fasting complement component 3 (C3)
- Mannose-binding lectin (MBL) activity
- Remnant-like particle cholesterol concentration (RLP-C)
- Parameters of cholesterol absorption and synthesis: plant sterols (campesterol and beta-sitosterol, reflecting cholesterol absorption) and lathosterol (reflecting cholesterol synthesis)
- Determination of atherogenic apo B-containing particles (VLDL, IDL and LDL subclasses),
- HDL particle phenotype and HDL particle composition
- Cholesterol ester transfer protein activity

12.3.2 Secondary efficacy analysis.

The combined cholesterol-lowering effect of treatment with *Plantago ovata husk* and statins to achieve the therapeutic objective will be studied. The proportion of successes, i.e. the patients in whom plasma LDL-C concentrations are below 130 mg/dl following combined treatment of *Plantago ovata husk* and statins will be calculated for each treatment.

The significance of the differences between these proportions will be evaluated using the chi-squares test. The null hypothesis considered will be that the proportion of success is the same in the two treatments for a two-tailed significance level of 0.05.

The effect of the treatment of *Plantago ovata* husk fibre on blood pressure will be evaluated by analytical procedures equivalent to those conducted to evaluate the effect of *Plantago ovata* husk fibre on plasma LDL-C concentration.

The effect of the combination of statins with *Plantago ovata* husk on blood pressure will be evaluated by analytical procedures equivalent to those conducted to evaluate the effect of the combination of statins and *Plantago ovata* husk on plasma LDL-C concentration.

The effect of the polymorphisms of apoliprotein E, the cholesterol ester transfer protein (CETP), apolipoprotein A-V and the FABP 2 on the response in plasma lipid concentrations will be made by adjusting multiple regression models over the difference in plasma LDL-C concentrations between visits.

Furthermore, possible variations in the effects of the soluble fibre and the statins according to the characteristics at the beginning of the study, LDL-C level, gender or BMI will be studied.

A multiple linear regression model will be adjusted over the difference in the plasma LDL-C concentrations between visit S8 and visit S0, where the treatment carried out and the endpoints of age, gender, BMI, or LDL-C level at visit S0 will be used as covariates.

A multiple linear regression model will be adjusted over the success proportion, i.e. patients in whom plasma LDL-C concentrations are below 130 mg/dl following the combined treatment of *Plantago ovata* husk and statins, where the treatment carried out and the endpoints of age, gender, BMI or level of LDL-C at visit S0 will be used as covariates.

12.3.3. Interim analyses

No interim analyses are provided for

12.3.4. Subgroup analysis

If the number of patients treated is high enough, subgroup comparisons will be conducted to evaluate whether there are changes in the values of plasma LDL-C concentrations between gender and for different initial LDL-C levels (130-149, 150-169, 170-189).
For this purpose, two-way ANOVAs will be performed [(treatment and gender) and (treatment, LDL-C start)] for the difference in plasma LDL-C concentrations between visit S8 and visit S0. The Bonferroni corrections will be taken into account for multiple post-hoc tests.

12.4.Analysis populations

The statistical analysis will be performed both for intention to treat (each patient is analysed in the group he/she was assigned to at the beginning of the study regardless of whether he/she did not comply with the corresponding intervention) and by protocol, which only includes patients who complied suitably with the intervention that was assigned to them.

The safety analysis will include all the patients who received study medication; the adverse events in each treatment will be described graphically and/or numerically.

The Spanish version 11 of the statistical application package SPSS will be used for the data analysis.

A two-tailed significance of 0.05 will be considered in all statistical tests.

12.4.Data management and Analysis centre

Quintiles: will be responsible of the management of the preparation and starting of the trial, as well as the monitoring of it. As well will be take care of the data management.

Asociación Hipótesis alternativa: Will be take care of the statstical analysis and the final report, as well statistic as clinical.

13. REFERENCES

1. Villar F, Banegas JR, de Mata J, Rodriguez-Artalejo F. Informe SEA 2003. Las enfermedades cardiovasculares y sus factores de riesgo en España: hechos y cifras. Sociedad Española de Arteriosclerosis. Fundación AstraZeneca.

2. Expert Panel on Detection, Evaluation, and Treatment of High Blood Cholesterol in Adults. Executive summary of the third report of the National Cholesterol Education Program (NCEP). *JAMA.* 2001;285:2486-2497.

3. Brotons C, Royo-Bordonada MA, Alvarez-Sala L, Armario P, Artigao R, Conthe P, de Alvaro F, de Santiago A, Gil A, Lobos JMª, Maiques A, Marrugat J, Mauricio D, Rodriguez-Artalejo F, Sans S, Suárez C, como Comité Español Interdisciplicario para la Prevención Cardiovascular (CEIPC). Spanish Adaptation of the European Guidelines for Cardiovascular Prevention. Publication pending by the journals of the scientific societies involved and the journal of the Ministry of Health and Consumer Affairs.

4. Goñi I. Dietary fibre intake in Spain: recommendations and actual consumption patterns. In Handbook of dietary fibre, edd SS Cho and ML Dreher, Chapter 39 New York. USA: Marcel-Dekker.

5. Saura-Calixto FD, García-Alonso A, Goñi I, Bravo L. *In vitro* determination of the indigestible fraction in foods: an alternative to dietary fiber analysis. J Agric Food Chem 2000; 48: 3342-334.

6. Saura-Calixto FD, Goñi I. The intake of dietary indigestible fraction in the Spanich diet shows the limitations of dietary fibre data for nutritional studies. Eur J Clin Nutr 2004; 58: 1078-1082.

7. MAPA Ministry of Agriculture, Fisheries and Food: La alimentación en España 2000. General Secretariat of Agriculture and Food, Madrid, Spain; 2001.

8. Enquesta d'alimentació i nutrició 2002-2003. Programa d'Alimentació i Nutrició de la Direcció General de Salut Pública. Autonomous Government of Catalonia.

9. Fernández ML. Soluble fiber and nondigestive carbohydrate effects on plasma lipids and cardiovascular risk. Curr Opin Lipidol 2001; 12: 35- 40.

10. Jenkins DJA, Kendall CWC, Axelsen M, Augustin LSA, Vuksan V. Viscous and nonviscous fibres, nonabsorbable and low glycemic index carbohydrates, blood lipids and coronary heart disease. Curr Opin Lipido 2000; 11:49-56.

11. Brown L, Rosner B, Willett WW, Sacks FM.Cholesterol-lowering effects of dietary fiber: a meta-analysis. Am J Clin Nutr 1999; 69:30-42.

12. Anderson JW, Allgood LD, Lawrence A, Altringer LA, Jerdack GR, Hergehold DA, Morel JG. Cholesterol-lowering effects of psyllium intake adjunctive to diet therapy in men and women with hypercholesterolemia: analysis of eight controlled trials. Am J Clin Nutr 2000; 71:1433-1438.

13. Ordovas JM, Mooser V. Nutrigenomics and nutrigenetics. Curr Opin Lipidol 2004; 15: 101-108.

14. Plan Integral de la cardiopatía isquémica 2004-2007. Ministry of Health and Consumer Affairs.

15. Lupton jR, Turner ND. Dietary fiber and coronary disease: Does the evidence support an Association? Curr Atheroscler Rep 2003; 5: 500-505.

16. Jula A, Marniemi J, Huuponen R et al. Effects of diet and simvastatin on serum lipids, insulin and antioxidants in hypercholesterolemic men-a randomized controlled trial. JAMA 2002, 287: 598-605.

17. Conroy RM, Pyöräla K, Fitzgerald AP, Sans S, Menotti A, de Backer G, et al. Estimation of ten-year risk of fatal cardiovascular disease in Europe: the SCORE project. Eur Heart J 2003; 24; 987-1003.

18. He J, Whelton PK. Effect of dietary fiber and protein intake on blood pressure: a review of epidemiologic evidence. Clin Exp Hypertens 1999, 21: 785-796.

14. SIGNATURE SHEET

Investigator's signature:

I have read and agree to carry out the study with code ***PLAN-EC-HIPERL-02***, "Multicentre, comparative, double-blind, two-arm parallel clinical trial of the effects of treatment with Plantago ovata husk on the lipid profile of patients with hypercholesterolemia". I am aware of my responsibilities as investigator and familiar with the study protocol. I agree to carry out the study and collaborate appropriately with the personnel in my team who participate in the study.

INVESTIGATOR: SIGNATURE:

POSITION: DATE:
AFFILIATION:

On behalf of the Sponsor:

NAME: Dr Anna Anguera Vilá SIGNATURE:

POSITION: Research Manager,
Madaus, S.A DATE:

ANNEX 1

Study evaluations diagram

STUDY EVALUATIONS DIAGRAM
VISITS

Day / Week	S-2	D-10	D-7	S0	S4	S7 (1)	S7 (2)	S8	S12	S15 (1)	S15 (2)	S16
Clinical history	X			X	X			X	X			X
Physical Examin.	X			X	X			X	X			X
Weight	X			X	X			X	X			X
Height	X											
BMI	X			X				X				X
Blood Pressure	X			X	X			X	X			X
Assessment Physical Activity	X							X				X
Informed Consent	X											
Lipid profile		X	X			X	X			X	X	
Genetic polymorphisms			X									
Biochemistry			X				X				X	
Haemogram			X				X				X	
Minerals			X				X				X	
Vitamins			X				X				X	
Delivery of diet record	X			X	X			X	X			
Collection of diet record			X		X		X		X		X	
Collection of AEs					X			X	X			X
Delivery of medication				X	X			X	X			
Collection of medication					X			X	X			X

ANNEX 2

Diet evaluations diagram

DIET EVALUATIONS DIAGRAM

	S-2	D-10	D-7	S0	S4	S5	S6	S7 (1)	S7 (2)	S8	S9	S10	S11	S12	13	S14	S15 (1)	S15 (2)	S16
**Delivery of diet record**	x			x	x					x				x					
**Collection of diet record**			x		x				x					x				x	
**24-h reminder**						x	x	x			x	x	x		x	x	x		

ANNEX 3

Patient Information Sheet

PATIENT INFORMATION SHEET

You have been invited to participate in a clinical trial. Before taking a decision you should understand the reason for this study and what your participation will involve for you. Please, read the following information carefully and decide freely if you want to participate.

The study will be conducted at the ___________________ Hospital by Dr. _________________ (name), whom you may contact on the telephone number _______________ if you have any question or any problems.

The consent form was revised and approved by the Hospital's Independent Ethics Committee (IEC).

Name of the study: ***Multicentre, comparative, double-blind, two-arm parallel clinical trial of the effects of treatment with Plantago ovata husk on the lipid profile of patients with hypercholesterolemia***

It has been demonstrated that high concentrations of cholesterol and low-density lipoprotein cholesterol (LDL-C, high risk cholesterol), are one of the major risk factors for coronary disease, myocardial infract and angina pectoris, all of which are the leading cause of death in the Western World.

Due to the great importance of this condition, both medically and socially, studies have been, and are being carried out to ascertain as best as possible what measures are needed to control the predisposing factors to these diseases, including lipid factors.

One of the newest measures for reducing plasma cholesterol levels is the intake of dietary fibre. The term dietary fibre defines a variety of substances present in plants, which resist the digestion of man's gastrointestinal enzymes.

The studies conducted hitherto to ascertain the effects of fibre on blood lipids have demonstrated that cholesterol can be reduced by consuming soluble fibre. Thus, the intake of this type of fibre in patients with high levels of LDL Cholesterol between 140 and 190 mg/dl leads to a reduction in this cholesterol. This effect has been quantified and there are data available with the use of a good example of soluble fibre, *Plantago ovata* husk.

The different studies conducted also show that the intake of soluble fibre is safe and well tolerated.

For all the above reasons, the improvement of blood levels of cholesterol, LDL cholesterol and HDL cholesterol (the so-called “good” cholesterol) deserves to be confirmed, with the intake of a specific type of soluble fibre, *Plantago ovata* husk, in patients presenting high levels of blood cholesterol. The addition of fibre could produce a synergic effect with the diet measures, and therefore increase the efficacy of a diet with a low fat content.

Consequently, the objective of this trial is to analyse the effects of fibre on LDL cholesterol in patients with LDL cholesterol higher than 140 mg/dl.

If you decide to participate in the study, you will first attend a screening visit to evaluate whether you meet the study inclusion criteria; following that, you will have a blood test (two extractions on two different days separated by 3-4 days) to determine your lipid profile. Once the results are available, and if you comply with the inclusion criteria, you will be randomly assigned to one of the two treatment groups:

- The group that will take soluble fibre or
- The group that will take placebo (substance without any effect).

You will continue with the diet defined from the start of the study.

The duration of the period of treatment with fibre will be 8 weeks.

If after 8 weeks you are seen to have reached the therapeutic objective established in the study in terms of reduction in LDL cholesterol, you will end the study at that point. However, if the levels of LDL have not yet fallen as expected, you will continue in the study, taking the same treatment but you will also be given another drug with an acknowledged lipoproteinemia lowering effect, a statin. You will follow this combined treatment for a further 8 weeks.

In view of the importance of diet compliance by the participants throughout the study, a dietician in each centre will be dedicated to the study from the preparation phase until the end of the study.

The following procedures will be implemented in order to evaluate your diet compliance and detect possible deviations from the defined recommendations:

- Diet records for 3 days, which will be delivered at the medical visits. You should complete these diet records in detail, following the instructions given by the dietician at all times. It is very important that you follow the instructions. If you have any questions, do not hesitate to talk to your dietician, who will be available on the following telephone number: ______________
- Similarly, 24-h diet reminders will be made by phone without notice to guarantee compliance.

At each control your dietician will ask your weight and you will be weighed at the visit indicated. In this way the effect of the diet on your body weight can be monitored.

Apart from the visits to your dietician, you will also have periodical visits to your doctor. In the course of these visits you will be asked about your state of health and will have a physical examination, and blood samples will also be taken at some of them for analysis, which will include, among others parameters, cholesterol levels. The blood samples will be taken in the week prior to the visit, on two different days separated by a 3-4 day interval. Furthermore, during the study your dietician or doctor will phone you to ask whether you are following the diet and taking your medication.

The study includes the extraction of DNA samples to perform genetic studies.

DNA is an element that is present in all cells, it is transmitted by your parents and bears a personalised code in the form of "genes", which make it possible to determine your personal physical characteristics, such as colour of the eyes, skin, etc. The differences between one person and another may help us to explain why some people respond better than others to treatment with fibre to reduce cholesterol levels.

You are also asked to give your consent for:

- *The DNA of your blood to be studied for some genes related to the metabolism of cholesterol and cardiovascular disease.*

According to the legislation in force, and it being a genetic study, the results obtained will not be reported to you or your doctor. The results will be analysed by groups or subgroups of participants. Total confidentiality is guaranteed with regard to the genetic data obtained from the study.

If you need to take any additional medication not recorded by the physician in charge of the study you should let him/her know in advance. During the study you cannot take drugs with blood glucose-lowering, cholesterol-lowering or weight-reducing action or any other dietary fibre-based preparations.

At each visit the physician will give you a new supply of the drug, sufficient to last until the next visit. At each visit you will be asked to return all containers (sachets/blisters), including used (empty) and/or unused ones in order to check compliance.

Your participation in the study is totally voluntary. You may refuse to participate or withdraw from the study at any time, with no penalty or without losing any benefits to which you are entitled.

Should you withdraw, neither your medical care nor your participation in other possible studies will be affected in any way whatsoever. The investigators may also terminate your participation without your consent.

Your participation in the study will be confidential, and all personal information that may come from the study will be treated with the utmost confidentiality in accordance with ethical standards and the legislation in force (Constitutional Law 15/1999 of 13 December, on personal data protection).

ANNEX 4

Informed Consent Form

INFORMED CONSENT

Multicentre, comparative, double-blind, two-arm parallel clinical trial of the effects of treatment with Plantago ovata husk on the lipid profile of patients with hypercholesterolemia

I...

Have read the information sheet that was given to me.

I was given the opportunity to ask questions on the study.

I have received sufficient information on the study.

I have been informed by Dr..

I understand that I can withdraw from the study:

- whenever I want
- without having to give any reasons
- without this affecting my medical care.

I freely give my consent to participate in this study.

Date: Participant's signature:

Date: Investigator's signature:

According to the Constitutional Law 15/1999 of 13 December, on personal data protection, your consent for the treatment of your personal data and for the transfer thereof can be revoked. You may use your right to access, change or cancel this data by contacting the investigator, who will inform the sponsor

ANNEX 5

Physical activity questionnaire

PHYSICAL ACTIVITY QUESTIONNAIRE

PHYSICAL ACTIVITY AND EXERCISE

Job / Occupation (8h) Type:____________

Almost always sitting down ☐
Sitting down half the time ☐
Almost always standing ☐
Almost always walking, lifts, carrying light loads ☐
Almost always walking, lifts, carrying heavy loads ☐
Heavy manual work ☐

Walking / bicycle (Not sport, mode of transport to get about, strolling, entertainment)

Hardly ever ☐
Less than 20 min/day ☐
20-40 min/day ☐
40-60 min/day ☐
1 – 1.5 h/day ☐
More than 1.5 h/day ☐

Home / housework (If it is not relevant, leave blank)

Less than 1 h/day ☐
1-2 h/day ☐
3-4 h/day ☐
5-6 h/day ☐
7-8 h/day ☐
More than 8h/day ☐

Free time (average day)
TV / Reading

Less than 1 h/day ☐
1-2 h/day ☐
3-4 h/day ☐
5-6 h/day ☐
More than 6 h/day ☐

Exercise (sports) **type: _____________**

Less than 1 h/week ☐
1 h/week ☐
2-3 h/week ☐
4-5 h/week ☐
More than 5 h/week ☐

How many hours a day do you sleep usually?	☐	hours
Habits (time taken to get dressed, shave, get ready,…)	☐	hours

Physical activity questionnaire. Assignment of values in MET

	MET values (not shown the questionnaire)
**Job / Occupation (8h) Type:____________**	
Almost always sitting down	1.3
Sitting down half of the time	1.8
Almost always standing	2.2
Almost always walking, lifts, doing a little carrying	2.6
Almost always walking, lifts, doing a lot of carrying	3.0
Heavy manual work	3.9
**Walking / bicycle**	3.6
(10’) Hardly ever (0.16)	
(15’) Less than 20 min / day (0.25)	
(30’) 20-40 min./day (0.50)	
(50’) 40-60 min./day (0.83)	
(75’) 1 – 1.5 h/day (1.15)	
(100’) More than 1.5 h/day (1.6)	
**Home / Housework**	2.5
(0.5h) Less than 1 h/day	
(1.5h) 1-2 h/day	
(3.5h)3 -4 h/day	
(5.5.h) 5-6 h/day	
(7.5h) 7-8 h/day	
(10h) More than 8h/day	
**Free time**	1.2
TV / Reading	
(0.5h) Less than 1 h/day	
(1.5h) 1-2 h/day	
(3.5 h) 3-4 h/day	
(5.5h) 5-6 h/day	
(8h) More than 6 h/day	
**Exercise**	5.0
(0.5h) Less than 1 h/week	
(1h) 1 h/week	
(2.5h) 2-3 h/week	
(4.5h) 4-5 h/week	
(6h) More than 5 h/week	
How many hours a day do you sleep usually? ☐ hours	0.9
Habits (time taken to get dressed, Shave, get ready,…) ☐ hours	2.0

*MET (metabolic equivalents), Kcal = METxKg

ANNEX 6

Food diary
(diet record)

Food diary

INSTRUCTIONS ON HOW TO COMPLETE THE FOOD DIARY

- We are interested in your usual food intake , so **please try not to change your eating habits**.

- Whenever you eat some food, you should **specify, as accurately as possible,** the type of food eaten. For example, you would have to specify:
  Milk: whole, skimmed or semi skimmed.
  Yoghurt: whole or skimmed, plain or fruit flavour, sweetened or enriched....
  Cheese: name of the cheese and its fat content (if known).
  Meat or fish: type of fish or meat (tuna, sole, hake or veal, chicken, lamb...), specifying whether it is loin, leg, breast, rib...
  Greens and cereals: the type of green or cereal, and whether they are fresh, frozen or tinned products.
  Bread: white or wholemeal bread.
  Fats and oils: butter, margarine, light margarine, cream, oil (specify the type of oil: olive, sunflower, corn...).
  Pre-cooked dishes, confectionery and biscuits: the name of the food.
  You can provide us with the nutritional composition or the list of ingredients described on the wrapping.
  Fruit: the name of the fruit and whether it is fresh or tinned.
  Drinks; the type of drink (low-calorie or not, with or without caffeine, natural or bottled juices, alcohol content, if necessary...).

- You should **specify the amount of each food type eaten**, taking, as your reference:
  - The specific weight on the package or wrapper, or as measured by you.
  - The number of spoonfuls.
  - The number of glasses, cups or bowls, after checking their capacity.
  - The number of units or parts of unit (for example, a packet of biscuits, half a packet, 5 biscuits).

- You should indicate any food or drink taken with meals and between them (sweets, chocolate, pastries, biscuits, juices, coffee...).

- You should specify, for each meal, **how they were cooked and the type of oil/fat used to make them** (olive or sunflower seed oil, margarine or butter, fried onion, garlic and tomato...).

- Remember to indicate the amount of sugar or honey added to yoghurt, coffee, tea or herbal drinks.

At the next visit we will check all the things described in the diary to make sure that you did not forget any food, as well as to clear up any doubts you may have.

FOOD RECORD

Patient's initials |___| |___|___|
N A

Randomisation No.|____|____|____|

1st day of diary Date |___|___|___|
D M Y

DETAILED DESCRIPTION OF MEALS AND DRINKS

DO NOT WRITE IN THESE COLUMNS!

**BREAKFAST AND ELEVENSES:** coffee, tea, milk, juices...	**Amount** (in grams or as measured at home)	**Food code**	**Name of food**	**Amount (g)**
**MEAL:** Remember to specify: food, amount, preparation or dressings, amount of dressings...				
**First course :** greens, cereals, potatoes, legumes...				
**Second course:** meat, fish, eggs...				
**Side-dish:** greens, mushrooms, potatoes, rice...				
**Dessert:** fruit, yoghurt, pastries, ice-cream...				
**Bread:** white, wholemeal bread...				
**Drinks:** water, fruit juices, beer, wine, coffee, tea...				

FOOD DIARY

Patient's initials |___| |___|___| (N A) **Randomisation No.|____|____|____|** **1st day of diary Date |___|___|___|** (D M Y)

Detailed description of meals and drinks | **DO NOT WRITE IN THESE COLUMNS!**

**DURING THE AFTERNOON:** coffee, tea, milk, juices...	**Amount** (in grams or as measured at home)	**Food code**	**Name of food**	**Amount (g)**
**EVENING MEAL:** Remember to specify: food, amount, preparation or dressings, amount of dressings...				
**First course:** greens, cereals, potatoes, legumes...				
**Second course:** meat, fish, eggs...				
**Side-dish:** greens, mushrooms, potatoes, rice...				
**Dessert:** fruit, yoghurt, pastries, ice-cream...				
**Bread:** white, wholemeal bread...				
**Drinks:** water, fruit juices, beer, wine, coffee, tea...				

COMMENTS

Patient's initials |___| |___|___|
N A

Randomisation No.|_____|_____|_____|

1st day of diary Date |___|___|___|
D M Y

ANNEX 7

24h diet reminder

24 HOUR REMINDER

Patient's initials |___| |___|___|　　No. Randomisation |____|____|　　Date of the reminder |___|___|___|　　Week ____
N　S　　　　　　　　　　　　　　　　　　　　　　　　　　　　　　　D　M　Y

BREAKFAST AND ELEVENSES	Food code	Name of food	Amount (g)
**MEAL**			
**DURING THE AFTERNOON**			
**EVENING MEAL AND NIGHT**			

ANNEX 8

Procedure Manual for collection, handling and storing of samples

Attached separately

ANNEX 9

Declaration of Helsinki

WORLD MEDICAL ASSOCIATION DECLARATION OF HELSINKI
Ethical Principles for Medical Research Involving Human Subjects

Adopted by the 18th WMA General Assembly, Helsinki, Finland, June 1964, and amended by the
29th WMA General Assembly, Tokyo, Japan, October 1975
35th WMA General Assembly, Venice, Italy, October 1983
41st WMA General Assembly, Hong Kong, September 1989
48th WMA General Assembly, Somerset West, Republic of South Africa, October 1996
and the 52nd WMA General Assembly, Edinburgh, Scotland, October 2000

A. INTRODUCTION

1. The World Medical Association has developed the Declaration of Helsinki as a statement of ethical principles to provide guidance to physicians and other participants in medical research involving human subjects. Medical research involving human subjects includes research on identifiable human material or identifiable data.

2. It is the duty of the physician to promote and safeguard the health of the people. The physician's knowledge and conscience are dedicated to the fulfillment of this duty.

3. The Declaration of Geneva of the World Medical Association binds the physician with the words, "The health of my patient will be my first consideration," and the International Code of Medical Ethics declares that, "A physician shall act only in the patient's interest when providing medical care which might have the effect of weakening the physical and mental condition of the patient."

4. Medical progress is based on research which ultimately must rest in part on experimentation involving human subjects.

5. In medical research on human subjects, considerations related to the well-being of the human subject should take precedence over the interests of science and society.

6. The primary purpose of medical research involving human subjects is to improve prophylactic, diagnostic and therapeutic procedures and the understanding of the aetiology and pathogenesis of disease. Even the best proven prophylactic, diagnostic, and therapeutic methods must continuously be challenged through research for their effectiveness, efficiency, accessibility and quality.

7. In current medical practice and in medical research, most prophylactic, diagnostic and therapeutic procedures involve risks and burdens.

8. Medical research is subject to ethical standards that promote respect for all human beings and protect their health and rights. Some research populations are vulnerable and need special protection. The particular needs of the economically and medically disadvantaged must be recognized. Special attention is also required for those who cannot give or refuse consent for themselves, for those who may be subject to giving consent under duress, for those who will not benefit personally from the research and for those for whom the research is combined with care.

9. Research Investigators should be aware of the ethical, legal and regulatory requirements for research on human subjects in their own countries as well as applicable international requirements. No national ethical, legal or regulatory requirement should be allowed to reduce or eliminate any of the protections for human subjects set forth in this Declaration.

B. BASIC PRINCIPLES FOR ALL MEDICAL RESEARCH

10. It is the duty of the physician in medical research to protect the life, health, privacy, and dignity of the human subject.

11. Medical research involving human subjects must conform to generally accepted scientific principles, be based on a thorough knowledge of the scientific literature, other relevant sources of information, and on adequate laboratory and, where appropriate, animal experimentation.

12. Appropriate caution must be exercised in the conduct of research which may affect the environment, and the welfare of animals used for research must be respected.

13. The design and performance of each experimental procedure involving human subjects should be clearly formulated in an experimental protocol. This protocol should be submitted for consideration, comment, guidance, and where appropriate, approval to a specially appointed ethical review committee, which must be independent of the investigator, the sponsor or any other kind of undue influence. This independent committee should be in conformity with the laws and regulations of the country in which the research experiment is performed. The committee has the right to monitor ongoing trials. The researcher has the obligation to provide monitoring information to the committee, especially any serious adverse events. The researcher should also submit to the committee, for review, information regarding funding, sponsors, institutional affiliations, other potential conflicts of interest and incentives for subjects.

14. The research protocol should always contain a statement of the ethical considerations involved and should indicate that there is compliance with the principles enunciated in this Declaration.

15. Medical research involving human subjects should be conducted only by scientifically qualified persons and under the supervision of a clinically competent medical person. The responsibility for the human subject must always rest with a medically qualified person and never rest on the subject of the research, even though the subject has given consent.

16. Every medical research project involving human subjects should be preceded by careful assessment of predictable risks and burdens in comparison with foreseeable benefits to the subject or to others. This does not preclude the participation of healthy volunteers in medical research. The design of all studies should be publicly available.

17. Physicians should abstain from engaging in research projects involving human subjects unless they are confident that the risks involved have been adequately assessed and can be satisfactorily managed. Physicians should cease any investigation if the risks are found to outweigh the potential benefits or if there is conclusive proof of positive and beneficial results.

18. Medical research involving human subjects should only be conducted if the importance of the objective outweighs the inherent risks and burdens to the subject. This is especially important when the human subjects are healthy volunteers.

19. Medical research is only justified if there is a reasonable likelihood that the populations in which the research is carried out stand to benefit from the results of the research.

20. The subjects must be volunteers and informed participants in the research project.

21. The right of research subjects to safeguard their integrity must always be respected. Every precaution should be taken to respect the privacy of the subject, the confidentiality of the patient's information and to minimize the impact of the study on the subject's physical and mental integrity and on the personality of the subject.

22. In any research on human beings, each potential subject must be adequately informed of the aims, methods, sources of funding, any possible conflicts of interest, institutional affiliations of the researcher, the anticipated benefits and potential risks of the study and the discomfort it may entail. The subject should be informed of the right to abstain from participation in the study or to withdraw consent to participate at any time without reprisal. After ensuring that the subject has understood the information, the physician should then obtain the subject's freely-given informed consent, preferably in writing. If the consent cannot be obtained in writing, the non-written consent must be formally documented and witnessed.

23. When obtaining informed consent for the research project the physician should be particularly cautious if the subject is in a dependent relationship with the physician or may consent under duress. In that case the informed consent should be obtained by a well-informed physician who is not engaged in the investigation and who is completely independent of this relationship.

24. For a research subject who is legally incompetent, physically or mentally incapable of giving consent or is a legally incompetent minor, the investigator must obtain informed consent from the legally authorized representative in accordance with applicable law. These groups should not be included in research unless the research is necessary to promote the health of the population represented and this research cannot instead be performed on legally competent persons.

25. When a subject deemed legally incompetent, such as a minor child, is able to give assent to decisions about participation in research, the investigator must obtain that assent in addition to the consent of the legally authorized representative.

26. Research on individuals from whom it is not possible to obtain consent, including proxy or advance consent, should be done only if the physical/mental condition that prevents obtaining informed consent is a necessary characteristic of the research population. The specific reasons for involving research subjects with a condition that renders them unable to give informed consent should be stated in the experimental protocol for consideration and approval of the review committee. The protocol should state that consent to remain in the research should be obtained as soon as possible from the individual or a legally authorized surrogate.

27. Both authors and publishers have ethical obligations. In publication of the results of research, the investigators are obliged to preserve the accuracy of the results. Negative as well as positive results should be published or otherwise publicly available. Sources of funding, institutional affiliations and any possible conflicts of interest should be declared in the publication. Reports of experimentation not in accordance with the principles laid down in this Declaration should not be accepted for publication.

C. ADDITIONAL PRINCIPLES FOR MEDICAL RESEARCH COMBINED WITH MEDICAL CARE

28. The physician may combine medical research with medical care, only to the extent that the research is justified by its potential prophylactic, diagnostic or therapeutic value. When medical research is combined with medical care, additional standards apply to protect the patients who are research subjects.

29. The benefits, risks, burdens and effectiveness of a new method should be tested against those of the best current prophylactic, diagnostic, and therapeutic methods. This does not exclude the use of placebo, or no treatment, in studies where no proven prophylactic, diagnostic or therapeutic method exists.

30. At the conclusion of the study, every patient entered into the study should be assured of access to the best proven prophylactic, diagnostic and therapeutic methods identified by the study.

31. The physician should fully inform the patient which aspects of the care are related to the research. The refusal of a patient to participate in a study must never interfere with the patient-physician relationship.

32. In the treatment of a patient, where proven prophylactic, diagnostic and therapeutic methods do not exist or have been ineffective, the physician, with informed consent from the patient, must be free to use unproven or new prophylactic, diagnostic and therapeutic measures, if in the physician's judgement it offers hope of saving life, re-establishing health or alleviating suffering. Where possible, these measures should be made the object of research, designed to evaluate their safety and efficacy. In all cases, new information should be recorded and, where appropriate, published. The other relevant guidelines of this Declaration should be followed.

ANNEX 10

Public liability policy

Attached separately

ANNEX 11

Standard Operating Procedures

MADAUS

CLINICAL INVESTIGATION

STANDARD OPERATING PROCEDURES

96.01	Organisational chart, definition of functions. Drafting, approval, distribution, archive, revision and update of investigation SOP. Training in investigation SOP
96.02	Application for Classification as Investigational New Drug
96.03	Preparation of the Investigator's Brochure
96.04	Preparation of protocol and CRF of a clinical trial
96.05	Informed Consent
96.06	Amendments to the protocol
96.07	Administrative management of a clinical trial: Contract, Insurance
96.08	Approval by IEC and Authorisation by AEM
96.09	Selection and recruitment of investigators/centres
96.10	Pre-study visit
96.11	Essential documents
96.12	Monitoring Manual
96.13	Investigators' Meetings
96.16	Assessment and prompt Notification of SAEs
96.17	Corrections of collected CRFs
96.19	CT Sample application, sending, labelling, storage and counting
96.20	CT file (Sponsor)
96.21	CT file (Investigator)
96.22	CT file on completion (Storage)
96.23	Annual Report to IECs, investigators, Autonomous Communities
96.24	Preparation, revision and approval of the CT Final Report
96.25	Management of subcontracting of CROs, centralised laboratory, phase I units, other services

The remaining procedures will follow the Quintiles' own SOPs.

ANNEX 13

Investigator's Brochure

Attached separately

ANNEX 14

Case Report Form

Attached separately

ANNEX 15

Economic report

Attached separately

ANNEX 16

Dietician's Manual

Attached separately
